# Modulating the Gut‐Microbiota‐Brain Axis in Alzheimer's Disease: Therapeutic Potential of Nutritional and Metabolic Factors

**DOI:** 10.1002/cns.71117

**Published:** 2026-08-30

**Authors:** Binyue Xu, Xuhuan Li, Shangling Dong, Zheyuan Zhang, Dong Jin, Guoping Li, Jiesheng Wang

**Affiliations:** ^1^ Department of Geriatric Medicine Tongde Hospital of Zhejiang Province Affiliated to Zhejiang Chinese Medical University Hangzhou China

**Keywords:** Alzheimer's disease, cognitive function, diet, gut microbiota, nutrition

## Abstract

**Background:**

Alzheimer's disease (AD) is a progressive neurodegenerative disorder and the leading cause of dementia among the elderly, characterized by a gradual decline in memory and cognitive function. The growing body of evidence highlighting the interaction between the gut microbiota and the central nervous system has positioned the gut microbiota as a key area of research in AD pathogenesis.

**Methods:**

This review critically evaluates the preclinical evidence and clinical trial outcomes, complemented by mechanistic studies and Mendelian randomization analyses, to assess the therapeutic potential of nutritional interventions targeting the gut‐microbiota‐brain axis in AD.

**Results:**

Dietary components and patterns regulate the composition and function of the gut microbiota, which in turn influence brain function through the gut‐microbiota‐brain axis via chemical/metabolic, immune‐mediated, and neural pathways. Specific nutrients, microbial metabolites, and dietary patterns have been shown to exert either protective or detrimental effects on AD pathology and cognitive function. Emerging strategies, including precision nutrition, fecal microbiota transplantation, and next‐generation microbiome‐based therapies, offer new avenues for AD prevention and treatment.

**Conclusions:**

Nutritional interventions targeting the gut‐microbiota‐brain axis represent a promising approach for the comprehensive prevention and management of AD. Further mechanistic and clinical studies are warranted to translate these findings into effective therapeutic strategies.

## Graphical abstract

This review systematically examines the role of gut microbiota modulation in Alzheimer's disease, focusing on how microbial metabolites and dietary nutrients influence cognitive function and AD pathology, as well as the neuroprotective effects through the gut‐brain axis. We further discuss future strategies, including precision nutrition, fecal microbiota transplantation, and next‐generation microbiome‐based therapies, as promising avenues for AD prevention and treatment.

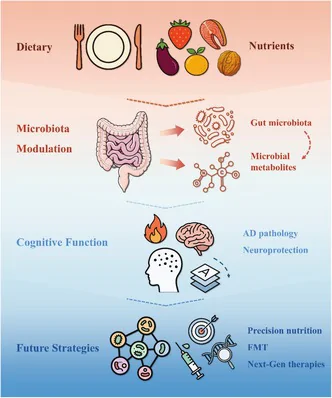

**Abbreviations**
5‐HT: 5‐hydroxytryptamine (serotonin)
AD: Alzheimer's disease
ADF: alternate‐day fasting
AhR: aryl hydrocarbon receptor
AI: artificial intelligence
APP/PS1: APPswe/PS1dE9 transgenic mouse model
ATRA: all‐trans retinoic acid
BBB: blood–brain barrier
CCN2: cellular communication network factor 2
CD4^+^
: cluster of differentiation 4 positive helper T cells
CD45: leukocyte common antigen
CDI: Clostridioides difficile infection
CNS: central nervous system
CSF: cerebrospinal fluid
DASH: Dietary Approaches to Stop Hypertension
DHA: docosahexaenoic acid
DPA: docosapentaenoic acid
EPA: eicosapentaenoic acid
FDA: U.S. Food and Drug Administration
FMO3: flavin monooxygenase 3
FMT: fecal microbiota transplantation
GABA: gamma‐aminobutyric acid
HIF1α: hypoxia‐inducible factor 1‐alpha
HPA: hypothalamic–pituitary–adrenal axis
IAA: indole‐3‐acetic acid
IF: intermittent fasting
IL: interleukin
ILA: indole‐3‐lactic acid
IPA: indole‐3‐propionic acid
KD: ketogenic diet
LC3B: microtubule‐associated protein 1A/1B‐light chain 3B
LPS: lipopolysaccharide
MCI: mild cognitive impairment
MCP: monocyte chemoattractant protein
MCTs: medium‐chain triglycerides
MeDi: Mediterranean diet
MHCII^+^
: major histocompatibility complex class II positive
MIND: Mediterranean‐DASH Intervention for Neurodegenerative Delay
MMSE: Mini‐Mental State Examination
Mo MDSC: monocytic myeloid‐derived suppressor cells
MoCA: Montreal Cognitive Assessment
MR: Mendelian randomization studies
mTOR: mammalian target of rapamycin
NF‐κB: nuclear factor kappa‐light‐chain‐enhancer of activated B cells
N‐3 PUFA: N‐3 polyunsaturated fatty acids
OmpA: outer membrane protein A
OMVs: outer membrane vesicles
P62: sequestosome 1
PF: periodic fasting
p‐Tau: phosphorylated tau
RCTs: randomized controlled trials
SAMP: senescence‐accelerated mouse‐prone
SCFAs: short‐chain fatty acids
SIRT1: sirtuin 1
SYK: spleen tyrosine kinase
SynComs: synthetic microbial communities
TGF: transforming growth factor
TLR: Toll‐like receptor
TMA: trimethylamine
TMAO: trimethylamine‐N‐oxide
TNF: tumor necrosis factor
TREM: triggering receptor expressed on myeloid cells
TRF: time‐restricted feeding
VFAs: volatile fatty acids

## 1 Introduction

As the world enters a deeply aging society, the prevalence of Alzheimer's disease (AD) is becoming increasingly severe. AD is expected to affect 131 million individuals globally by the year 2050 [1]. Studies indicate that the prevalence of AD doubles every 5 years as age increases [2]. This increasing prevalence poses major threats to the physical and mental health as well as the quality of life of the elderly population, emerging as a significant public health challenge at present. The pathogenesis of AD is multifactorial, complex, and not yet fully elucidated, involving genetic predisposition, lifestyle factors, and environmental influences, among others. Existing research indicates that the characteristic pathological hallmarks of AD primarily include the abnormal extracellular deposition of amyloid‐β (Aβ), forming senile plaques, and the hyperphosphorylation of the microtubule‐associated protein tau, leading to the formation of neurofibrillary tangles and neuronal loss within neurons [3]. These pathological changes are accompanied by gliosis, which further contributes to the progression of AD.

Despite the proliferation of research on AD‐related mechanisms, there is still no complete cure for AD, and the need to prevent or delay AD and mild cognitive impairment (MCI) progression remains unmet. Therefore, the management of potential risk factors is currently considered the most effective approach for preventing dementia, AD, and MCI [4, 5, 6]. Both clinical research and expert consensus have indicated that nutritional intervention plays an important role in maintaining brain health, modulating risk factors, delaying disease progression, and improving overall prognosis [7, 8]. The 2011 National Institute on Aging‐Alzheimer's Association (NIA‐AA) guidelines demonstrated a direct relationship between diet and changes in brain structure and activity [9]. The potential role of diet in preventing or slowing neurodegeneration or enhancing certain cognitive abilities has thus become a major focus of research in both preclinical stages and clinical research.

As the roles of gut microbiota in aging, immunity, and metabolism continue to be elucidated, numerous studies have found that gut microbiota dysbiosis is prevalent in AD‐related cognitive impairment [10, 11]. Consequently, the involvement of gut microbiota composition and its metabolites in the pathogenesis of AD development through the “Gut‐Microbiota‐Brain Axis” has become a major research focus in recent years, also providing novel directions and approaches for AD therapeutic strategies. Research indicates that diet and specific nutrients can influence the composition of the gut microbial community [12] and amyloid production or aggregation [13, 14]. Modulating the gut microbiota through specific nutritional interventions and the use of probiotics, fecal microbiota transplantation (FMT) and next‐generation microbiome‐based therapies may effectively reduce AD‐associated chronic inflammation and Aβ levels, thereby preventing or ameliorating AD symptoms.

## 2 Methods

Relevant literature was retrieved from the PubMed and Web of Science, covering the publication period from database inception to February 2026. This review included clinical trials, observational studies, and experimental studies conducted on animal models that explored the mechanistic links between nutritional interventions, gut microbiota modulation, and AD pathology. Studies focusing on MCI, prodromal AD, or preclinical stages of AD were also considered for inclusion, as these populations represent critical windows for nutritional interventions. Additionally, the reference lists of published systematic reviews and meta‐analyses were manually screened to identify additional eligible original studies not captured in the initial database searches.

The search strategy incorporated the following keywords across three thematic domains, combined using Boolean operators (“AND” and “OR”), including but not limited to: 1. Alzheimer's disease and related cognitive decline: “Alzheimer's disease,” “mild cognitive impairment,” “MCI,” “amyloid beta,” “Aβ,” “tau protein.” 2. Gut microbiota: “gut microbiota,” “gut microbiome,” “gut microflora,” “microbiota‐gut‐brain axis,” “Bacteroidetes,” “Firmicutes,” “Proteobacteria.” 3. Nutritional interventions: “nutritional intervention,” “short‐chain fatty acids,” “n‐3 polyunsaturated fatty acids,” “polyphenols,” “trimethylamine‐N‐oxide,” “dietary patterns,” “Mediterranean diet,” “ketogenic diet,” “intermittent fasting.”

## 3 Gut‐Microbiota‐Brain Axis in AD

The human gut microbiota is predominantly composed of five bacterial phyla: *Firmicutes*, *Bacteroidetes*, *Actinobacteria*, *Proteobacteria*, and *Verrucomicrobia* [15]. Among these, Bacteroidetes and Firmicutes constitute the majority of the gut bacterial community, while Proteobacteria and Actinobacteria are present in smaller proportions [16]. Accumulating evidence indicates that patients with cognitive impairment and cerebral amyloidosis exhibit alterations in gut microbiome composition [17, 18], suggesting a link between gut dysbiosis and neuroinflammation. Mechanistically, gut dysbiosis is thought to promote intestinal barrier disruption, facilitate the translocation of bacterial products, and ultimately influence Aβ metabolism and deposition in the brain [19, 20].

### 3.1 Bacteroidota

*Bacteroidota*, particularly the genus *Bacteroides*, are Gram‐negative obligate anaerobes that ferment carbohydrates to produce volatile fatty acids (VFAs), which serve as a major energy source for colonocytes and modulate intestinal homeostasis [21, 22, 23, 24]. Their abundance is strongly influenced by diet: an animal‐based or high‐fat/low‐fiber diet increases *Bacteroides* levels, whereas a plant‐based diet is associated with lower abundance [25, 26].

Studies have reported lower Bacteroides abundance in AD patients [17, 27, 28], likely due to its protective role: its certain metabolites such as acetate and propionate inhibit pro‐inflammatory cytokine release, maintain intestinal barrier and blood–brain barrier (BBB) integrity, and suppress Aβ deposition [29, 30]. Beyond VFAs, *Bacteroidota*‐derived serine‐glycine (S/G) lipids have been shown to suppress monocyte proinflammatory responses by maintaining expression of NF‐κB and TLR pathway inhibitors, including Trem2, a mechanism that may also be relevant to microglial function in AD [31]. Within the same phylum, increased abundance of *Parabacteroides* has also been shown to reduce AD risk by elevating serum alanine levels [32].

Conversely, under conditions of increased gastrointestinal and BBB permeability, which are common in aging and AD, *Bacteroides*‐derived lipopolysaccharide (LPS) and proteolytic peptides can impair microglia‐mediated innate immunity, phagocytosis, and detoxification, while also promoting Aβ production, thereby sustaining chronic and progressive neuroinflammation [11, 33, 34, 35]. In vivo studies confirm that oral administration of *Bacteroides fragilis* suppresses microglial Aβ clearance [36] and activates microglia in neuronal C/EBPβ transgenic mice [37, 38]. These discrepant findings may arise from differences in sample size, study design, host species, confounding factors, and interactions with other microbial communities.

Notably, a longitudinal study in AD mice revealed that *Bacteroides* abundance increased over time, with compositional differences detectable as early as 8 weeks of age, which precedes the onset of cognitive deficits and amyloid pathology. This temporal pattern suggests that the direction of microbial change may depend on disease stage and that early microbiota alterations may precede overt pathology, adding further complexity to the interpretation of cross‐sectional findings [39]. The dual roles of *Bacteroidetes* in AD [40], together with their known responsiveness to diet, raise the possibility that dietary modulation could influence AD pathogenesis. Future research integrating multi‐omics approaches and longitudinal tracking is needed to resolve current contradictions and identify strain‐specific therapeutic targets.

### 3.2 Firmicutes

*Firmicutes* play a crucial role in maintaining host homeostasis. Many members of this phylum, including *Bacillus* and *Lactobacillus*, are widely recognized as probiotics [41]. *Firmicutes* utilize dietary fiber to produce short‐chain fatty acids (SCFAs) such as acetate, butyrate, and lactate [42], which exert beneficial effects on cognition through anti‐inflammatory, antioxidant, and insulin‐sensitizing mechanisms [43, 44].

Multiple cohort studies have consistently reported a reduced proportion of *Firmicutes* in AD patients [45, 46, 47]. Within this phylum, genera such as *Roseburia* and *Faecalibacterium* have been positively correlated with MMSE and MoCA scores [45]. Similarly, the anti‐inflammatory species *Eubacterium rectale* (class *Clostridia*) shows decreased abundance in cognitively impaired individuals with cerebral amyloidosis [17]. Animal studies have confirmed the neuroprotective potential of multiple *Firmicutes* species through diverse mechanisms, including SCFA modulation, regulation of Tau phosphorylation, suppression of neuroinflammation, activation of antioxidant defenses, and direct inhibition of Aβ aggregation [48, 49, 50, 51, 52, 53, 54, 55, 56, 57, 58, 59].

Notably, not all *Firmicutes* species uniformly decline. *Ruminococcus gnavus* was enriched in older adults with cognitive impairment and inversely correlated with MMSE scores. Monocolonization of aged pseudo‐germ‐free mice with *R. gnavus* impaired learning and memory, accompanied by suppressed autophagy markers (decreased LC3B and Beclin‐1, increased P62) [60]. Likewise, *Blautia coccoides* and *Lactobacillus murinus* have also been associated with exacerbated AD pathology [61, 62], suggesting that species‐specific effects within the same phylum may oppose the general protective trend. The association between *Firmicutes* and cognitive function, together with their responsiveness to dietary fiber, supports the potential of fiber‐based nutritional strategies to modulate this phylum. Future studies are needed to clarify the specific roles of different *Firmicutes* species and their underlying mechanisms.

### 3.3 Proteobacteria

The *Proteobacteria* phylum comprises a diverse assemblage of Gram‐negative bacteria, including the families *Enterobacteriaceae* (*Escherichia*, *Klebsiella*), *Desulfovibrionaceae*, and *Vibrionaceae*, as well as the family *Helicobacteraceae* (e.g., the gastric colonizer *Helicobacter pylori* ). Through their predominantly pro‐inflammatory properties, members of this phylum have been implicated in the pathogenesis of neurodegenerative disorders via the gut‐brain axis. Multiple cross‐sectional studies have consistently reported an elevated abundance of *Proteobacteria* in AD patients. *Gammaproteobacteria* and *Enterobacteriales* progressively enriched from healthy controls to prodromal MCI and AD stages [45]. Notably, cognitively normal individuals with cerebral Aβ deposition (CN+) exhibited a significantly lower abundance of class *Deltaproteobacteria* compared to those without Aβ deposition (CN−), suggesting that alterations in *Proteobacteria* subclasses may precede clinical symptoms [63].

Mechanistic evidence from animal models supports a causal role for multiple *Proteobacteria* species. *Enterobacteria* infection exacerbated neurodegeneration in a Drosophila AD model [64]. In mouse experiments, several *Proteobacteria* species have been shown to exacerbate AD‐related pathology and cognitive decline through mechanisms involving microbial translocation, immune activation, and microglial dysfunction [65, 66, 67, 68]. Outer membrane vesicles (OMVs) derived from *Helicobacter pylori* (*Hp*) have been shown to cross biological barriers and enter the brain, leading to glial activation, neuronal dysfunction, and exacerbated Aβ pathology [69]. The translational relevance of these findings is underscored by direct evidence from human brain tissues. LPS derived from *Escherichia coli* is enriched in the hippocampus and neocortex, where it colocalizes with Aβ1–40/42 within amyloid plaques. Additionally, *E. coli* K99 pili protein was detected specifically in neuron‐like cells, suggesting that bacterial components can access the brain parenchyma and participate in plaque‐associated pathology [70, 71].

However, strain‐level heterogeneity exists within this phylum: the probiotic *Escherichia coli* Nissle 1917 exerted protective effects via outer membrane protein A (OmpA)‐containing OMVs that were internalized by glia and neurons, suppressing glial hyperactivation and restoring synaptic function in APP/PS1 mice. These findings provide insights into how probiotic‐derived OMVs modulate neuroimmune crosstalk in the CNS, establishing a mechanistic foundation for developing OMV‐based therapeutic strategies for AD [72].

### 3.4 Mendelian Randomization Studies (MR)

Observational studies have consistently reported associations between gut microbiota composition and AD. However, findings across studies are often inconsistent [73, 74], probably due to (1) confounding factors, such as diet, medications (e.g., antibiotics, proton pump inhibitors), and lifestyle; (2) reverse causation, as AD‐related pathological or behavioral changes may alter gut microbiota rather than the reverse; (3) methodological heterogeneity, including differences in 16S rRNA sequencing platforms, bioinformatic pipelines, and taxonomic classification; (4) small sample sizes; and (5) population restriction to single ethnic or geographic groups [75, 76]. Consequently, it remains unclear whether the observed associations reflect causal relationships.

MR provides a complementary strategy. By using genetic variants as instrumental variables for modifiable exposures, MR is less susceptible to confounding and reverse causation and has been widely used to investigate causal relationships between exposures and outcomes [77]. As summarized in Table 1, multiple MR studies suggest that specific gut microbial taxa exert causal effects on AD risk. This genetic evidence supports the investigation of microbiome‐targeted nutritional interventions as a potential strategy for AD.

**TABLE 1:** Causal relationships between gut microbiota and AD in Mendelian randomization studies.

Phylum	Microbiota	Ranks	Outcome	Ref.
*Actinobacteria*	*Actinobacteria*	Phylum	Increase	[78, 79]
	*Actinobacteria*	Class	Increase	[78, 80, 81, 82, 83, 84, 85, 86, 87]
	*Actinobacteriota*	Phylum	Decrease	[88]
	*Actinomycetales*	Order	Increase	[79]
	*Bifidobacteriaceae*	Family	Increase	[89]
	*Bifidobacteriales*	Order	Increase	[89]
	*Bifidobacterium*	Genus	Increase	[79, 90]
	*Bifidobacterium*	Genus	Decrease	[91]
	*Bifidobacterium adolescentis*	Species	Increase	[79]
	*Bifidobacterium angulatum*	Species	Increase	[79]
	*Bifidobacterium bifidum*	Species	Decrease	[80, 85]
	*Bifidobacterium infantis*	Species	Decrease	[88]
	*Bifidobacterium kashiwanohense*	Species	Increase	[79]
	*Bifidobacterium ruminantium*	Species	Increase	[79]
	*Coriobacteriaceae*	Family	Increase	[80, 85]
	*Coriobacteriales*	Order	Increase	[80, 85, 92]
	*Coriobacteriia*	Class	Ns	[87]
	*Corynebacterium*	Genus	Decrease	[79]
	*Eggerthella*	Genus	Decrease	[80, 85, 93]
	*Slackia*	Genus	Decrease	[91, 94]
*Bacteroidota*	*Alloprevotella*	Genus	Decrease	[32]
	*Bacteroidaceae*	Family	Decrease	[80, 85, 92]
	*Bacteroides*	Genus	Decrease	[32, 80, 85]
	*Bacteroides sp002160055*	Species	Increase	[88]
	*Bacteroidia*	Class	NS	[87]
	*Parabacteroides*	Genus	Decrease	[32]
	*Parabacteroides johnsonii*	Species	Decrease	[79]
	*Paraprevotella*	Genus	Decrease	[91]
	*Prevotella*	Genus	Increase	[32, 90]
*Firmicutes*	*Acidaminococcus fermentans*	Genus	Increase	[88]
	*Agathobacter*	Genus	Decrease	[90]
	*Allisonella*	Genus	Increase	[89, 95]
	*Allisonella*	Genus	Decrease	[96]
	*Anaerotruncus*	Genus	Decrease	[80, 85, 95]
	*Bacillales*	Order	Decrease	[89, 95, 97]
	*Bacilli*	Class	Decrease	[80, 85]
	*Bacilli*	Class	NS	[87]
	*Bacillus*	Genus	Decrease	[79]
	*Bacillus Velezensis*	Species	Decrease	[79]
	*Blautia*	Genus	Decrease	[98]
	*Butyrivibrio*	Genus	Decrease	[80, 85]
	*CAG‐177 sp002451755*	Species	Decrease	[88]
	*CAG‐194*	Genus	Decrease	[79]
	*CAG‐194 sp002441865*	Species	Decrease	[79]
	*CAG‐269 sp002372935*	Species	Increase	[88]
	*CAG‐349*	Genus	Decrease	[79]
	*CAG‐449*	Genus	Increase	[79]
	*CAG‐465 sp000433135*	Species	Decrease	[88]
	*CAG‐485 sp002362485*	Species	Decrease	[79]
	*CAG‐488*	Species	Decrease	[88]
	*CAG‐81 sp000435795*	Species	Decrease	[79]
	*CAG‐81 sp000435795*	Species	Increase	[88]
	*CAG‐826*	Genus	Increase	[79]
	*CHKCI006 sp900018345*	Species	Decrease	[79]
	*Clostridia*	Class	Decrease	[80, 85, 87]
	*Clostridiales*	Order	Decrease	[80, 85]
	*Clostridium asparagiforme*	Species	Decrease	[80, 85]
	*Clostridium E sporosphaeroides*	Species	Decrease	[90]
	*Clostridium innocuum group*	Genus	Decrease	[80, 85, 93]
	*Clostridium M clostridioforme*	Species	Increase	[79]
	*Clostridium P*	Genus	Decrease	[88]
	*Defluviitaleaceae*	Family	Decrease	[95]
	*Defluviitaleaceae UCG011*	Genus	Increase	[91, 96]
	*Dialister*	Genus	Increase	[84]
	*Dorea*	Genus	Increase	[99]
	*Erysipelotrichia*	Class	Ns	[87]
	*Eubacteriumbrachygroup*	Species	Decrease	[83]
	*Eubacterium callanderi*	Species	Decrease	[79]
	*Eubacterium Q*	Genus	Decrease	[88]
	*Eubacterium R*	Genus	Decrease	[90]
	*Eubacterium ventriosum group*	Genus	Increase	[91]
	*Eubacterium_hallii*	Species	Increase	[80, 92]
	*Faecalibacterium*	Genus	Decrease	[81]
	*Holdemanella*	Genus	Increase	[32, 83]
	*Holdemania massiliensis*	Species	Increase	[90]
	*Intestinimonas*	Genus	Decrease	[80, 85, 89]
	*Intestinimonas*	Genus	Increase	[96]
	*Intestinimonas massiliensis*	Species	Decrease	[79]
	*Intestinimonas massiliensis*	Species	Increase	[90]
	*KLE1615*	Genus	Increase	[79]
	*Lachnoclostridium*	Genus	Increase	[81, 82]
	*Lachnospiraceae*	Family	Decrease	[32, 79]
	*Lachnospiraceae*	Genus	Decrease	[80]
	*Lachnospiraceae FCS020*	Genus	Decrease	[96]
	*Lachnospiraceae FCS020 group*	Genus	Increase	[89, 95]
	*Lachnospiraceae (NK4A136 group)*	Genus	Decrease	[91, 94]
	*LachnospiraceaeUCG004*	Genus	Decrease	[85]
	*Lactobacillaceae*	Family	Increase	[80, 81, 82, 84, 85]
	*Lactococcus*	Genus	Increase	[83]
	*Lawsonibacter sp900066645*	Species	Increase	[79]
	*Megamonas funiformis*	Species	Decrease	[90]
	*Megasphaera*	Genus	Increase	[90]
	*Negativicutes*	Class	Increase	[80, 85]
	*Oscillibacter*	Genus	Decrease	[80, 92, 96]
	*Oscillibacter_unclassified*	Species	Decrease	[92]
	*Oscillospira*	Genus	Increase	[78, 83]
	*Oscillospiraceae*	Family	Decrease	[80, 92]
	*Paenibacillus J*	Genus	Increase	[90]
	*Phocea*	Genus	Decrease	[88]
	*RUG472 sp900319345*	Species	Increase	[79]
	*Ruminiclostridium 6*	Genus	Increase	[81, 82]
	*Ruminiclostridium 9*	Genus	Decrease	[80, 81, 82, 85, 93]
	*Ruminococcaceae UCG‐003*	Genus	Increase	[91]
	*Ruminococcaceae UCG004*	Genus	Increase	[78]
	*Ruminococcus*	Genus	Decrease	[32]
	*Ruminococcus1*	Genus	Decrease	[78, 83]
	*Selenomonadales*	Order	Increase	[80, 85, 93]
	*Sellimonas*	Genus	Increase	[95, 97]
	*Turicibacter*	Genus	Increase	[79, 99]
	*Turicibacter sp001543345*	Species	Decrease	[79]
	*Turicibacteraceae*	Family	Decrease	[79]
	*UBA2821*	Species	Increase	[88]
	*UBA3792*	Species	Decrease	[88]
	*UBA6382*	Species	Increase	[88]
	*UBA9475 sp002161235*	Species	Increase	[79]
	*Veillonella*	Genus	Decrease	[89]
	*Veillonella unclassified*	Species	Increase	[80, 85]
*Proteobacteria*	*Alphaproteobacteria*	Class	NS	[87]
	*An7*	Unclassified	Increase	[88]
	*Aureimonas*	Genus	Decrease	[79]
	*Azorhizobium*	Genus	Decrease	[88]
	*Betaproteobacteria*	Class	Decrease	[83]
	*Betaproteobacteria*	Class	Increase	[80, 85, 92]
	*Betaproteobacteria*	Class	NS	[87]
	*Burkholderiales*	Order	Increase	[80, 85, 92]
	*Citrobacter A*	Genus	Decrease	[90]
	*Deltaproteobacteria*	Class	Increase	[78, 83]
	*Deltaproteobacteria*	Class	NS	[87]
	*Desulfovibrio*	Genus	Increase	[91, 94]
	*Desulfovibrionaceae*	Family	Increase	[76, 83, 97, 99]
	*Desulfovibrionales*	Order	Increase	[76, 78, 83, 97, 99]
	*Enterobacteriaceae*	Family	Decrease	[89]
	*Enterobacteriales*	Order	Decrease	[89]
	*Gammaproteobacteria*	Class	NS	[87]
	*Hydrogenophaga*	Genus	Increase	[90]
	*Klebsiella A*	Genus	Decrease	[88]
	*Klebsiella pneumoniae*	Genus	Decrease	[88]
	*Pasteurellaceae*	Family	Increase	[80, 85, 93]
	*Pasteurellales*	Order	Increase	[80, 85]
	*Pseudomonas aeruginosa*	Species	Decrease	[90]
	*Raoultella*	Genus	Increase	[90]
	*Sutterella*	Genus	Decrease	[32]
	*Sutterellaceae*	Family	Increase	[80, 85]
*Chlamydiota*	*Parachlamydiales*	Genus	Increase	[88]
*Spirochaetota*	*Borreliaceae*	Order	Decrease	[88]
	*Treponema D*	Genus	Decrease	[88]
*Tenericutes*	*Tenericutes*	Phylum	Decrease	[80]
	*Mollicutes*	Class	Decrease	[80, 85, 93]
	*Haloplasmatales*	Order	Decrease	[79]
*Verrucomicrobiota*	*Roseibacillus*	Genus	Decrease	[88]
*Archaea‐Euryarchaeota*	*Methanobrevibacter*	Genus	Increase	[80, 93]
*Undefined*	*AR31*		Increase	[79]

*Note:* This table summarizes Mendelian randomization studies that have investigated causal relationships between gut microbiota and AD. For each microbial taxon, we have included its phylum classification, taxonomic rank, and reported association with AD risk (i.e., whether it increases or decreases the risk of AD).

### 3.5 Gut‐Brain Signaling Pathways

Gut dysbiosis is increasingly recognized as a contributing factor in AD pathogenesis. The gut‐brain signaling network involves multiple interacting mechanisms that collectively link microbial imbalance to neurodegeneration (Table 2). Dysbiosis can compromise intestinal barrier integrity, facilitating the translocation of microbial products into systemic circulation [101]. These microbial signals, including both bioactive metabolites and structural components, influence neural function through distinct routes.

**TABLE 2:** Effects of gut microbial taxa on Alzheimer's disease pathology in experimental studies.

No.	Study type	Study subjects	Gut micro‐organism	Phylum	Effect on AD	Mechanism	Ref.	Year
1	Animal study	APP/PS1‐21 mice	*Bacteroides fragilis*	*Bacteroidota*	Detrimental	Suppresses microglial phagocytic function → impaired Aβ clearance → Aβ plaque accumulation	[36]	2024
2	Animal study	Thy1‐human C/EBPβ transgenic mouse; AD patient FMT	*Bacteroides fragilis*	*Bacteroidota*	Detrimental	*B. fragilis* and its PUFA metabolites (12‐HHTrE, PGE2) activate microglia → upregulate C/EBPβ/AEP pathway → induce AD pathologies	[37]	2023
3	Animal study	5xFAD mouse	*Bacteroides ovatus*	*Bacteroidota*	Protective	*B. ovatus* hydrolyzes PC to LPC via PLA2; LPC activates GPR119 → upregulates NRF2 → inhibits ACSL4 expression → suppresses ferroptosis → reduces Aβ plaques, rescues synaptic function, decreases gliosis, ameliorates myelin degeneration	[100]	2025
4	Animal study	Germ‐free 3 × Tg mice; AD patient FMT	*Bacteroides intestinalis* , *B. fragilis* , *B. xylanisolvens*	*Bacteroidota*	Detrimental	Activates C/EBPβ/AEP pathway → upregulates PUFA metabolites and oxidative enzymes → microglia activation and neuroinflammation → exacerbates Aβ plaques, neurofibrillary tangles, and cognitive dysfunctions	[38]	2022
5	Animal study	*Caenorhabditis elegans*	*Bacteroides rodentium*	*Bacteroidota*	Detrimental	Increases Aβ plaques; suppresses p38‐MAPK activation; exacerbates paralysis	[51]	2025
6	Animal study	5xFAD transgenic AD mouse	*Clostridium sporogenes* *+ xylan* (synbiotic)	*Firmicutes*	Protective	Synbiotic ( *C. sporogenes* + xylan) enhances tryptophan metabolism → increases IPA production and IPA‐synthesizing bacteria (*Lachnospira*, *Clostridium*) → restores synaptic ultrastructure, suppresses neuroinflammation, reduces Aβ deposition in cortex and hippocampus, maintains gut barrier integrity	[49]	2024
7	Animal study + Cell experiment	APP/PS1 mice; microglial cells	*Clostridium butyricum*	*Firmicutes*	Protective	Increases acetate‐producing bacteria and SCFAs; upregulates tight junction proteins (Claudin‐1, ZO‐1, Occludin) to restore intestinal barrier and reduce serum LPS; suppresses neuroinflammation (IL‐6, TNF‐α, IL‐1β) in brain; regulates JNK/CDK5/GSK‐3β pathway to inhibit Tau hyperphosphorylation; reduces neuronal apoptosis (Bax/Bcl‐2); acetate inhibits JAK/STAT3 signaling in microglia, reducing Aβ and p‐Tau	[50]	2025
8	Animal study	*Caenorhabditis elegans*	*Limosilactobacillus reuteri*	*Firmicutes*	Protective	Reduces Aβ plaque burden in head region; activates p38‐MAPK pathway (upregulates Tol‐1, Pmk‐1, Skn‐1); reduces paralysis rate	[51]	2025
9	Animal study	5xFAD mice	*Lactobacillus suilingensis + inulin (synbiotic)*	*Firmicutes*	Protective	Synbiotic enhances tryptophan metabolism → increases ILA biosynthesis → ILA activates AhR signaling pathway in the brain → inhibits neuroinflammation → reduces Aβ accumulation and alleviates cognitive impairment	[52]	2025
10	Animal study + Cell experiment	5xFAD mice; cell culture with aryl hydrocarbon receptor (AhR) inhibitor	*Lactobacillus reuteri*	*Firmicutes*	Protective	ILA activates AhR signaling in the brain → activates microglia and astrocytes → reduces soluble Aβ levels → prevents Aβ accumulation and cognitive impairment	[53]	2024
11	Animal study + Cell experiment	Male adult MAPT P301S transgenic mice; Neuro‐2a/HEK‐293 T cells	*Blautia coccoides*	*Firmicutes*	Detrimental	Produces TMAO; TMAO directly binds HIF1α → inhibits HIF1α signaling (HIF1α, HO‐1, SOD2, GPX4↓) → promotes oxidative stress (ROS↑) → promotes Tau phosphorylation (p‐Tau181, p‐Tau396↑) → exacerbates cognitive impairment	[61]	2025
12	Animal study + Cell experiment	5xFAD transgenic mice; human intestinal epithelial cells (HT29)	*Lactobacillus murinus*	*Firmicutes*	Detrimental	*L.m*. adheres to gut epithelia → increases lactate production → lactate activates GPR81‐NFκB axis in epithelial cells → stimulates SAA production → activates monocytes/DCs → promotes peripheral Th1 inflammation → exacerbates AD pathology	[62]	2024
13	Animal study	Male C57BL/6 mice	*Lacticaseibacillus casei*	*Firmicutes*	Protective	Modulates Akt/CREB/BDNF signaling pathway → reduces Aβ aggregation, inhibits Tau hyperphosphorylation, prevents neuronal death → improves cognitive dysfunction	[54]	2022
14	Animal study	D‐galactose/AlCl3 induced AD rats	*Lactobacillus plantarum MA2*	*Firmicutes*	Protective	*MA2* alleviates intestinal mucosal impairment → inhibits microglial activation via TLR4/MYD88/NLRP3 signaling pathway → *MA2* reduces neuroinflammation and Aβ accumulation → improves cognitive deficits; *MA2*‐derived EPS directly inhibit Aβ42 aggregation and amyloid‐induced cytotoxicity	[55]	2022
15	Animal study	D‐galactose and AlCl3‐induced AD model mice	*Lactiplantibacillus plantarum*	*Firmicutes*	Protective	Regulates PI3K/Akt/GSK‐3β pathway → inhibits Tau hyperphosphorylation; increases serotonin, dopamine, and GABA levels; ameliorates neuronal damage, Aβ deposition, and Tau pathology; modulates gut microbial communities → improves cognitive function	[56]	2022
16	Animal study	APP transgenic mice	*Lactiplantibacillus plantarum LAB12 and Lacticaseibacillus casei Shirota*	*Firmicutes*	Protective	Downregulates BACE1 mRNA expression; increases glutathione; reduces nitric oxide; improves memory	[57]	2023
17	Animal study	Male APP/PS1 mice	*Limosilactobacillus mucosae*	*Firmicutes*	Protective	Activates CB2 → upregulates Nrf2/HO‐1 antioxidant pathway → increases SOD2 and GPX4 → reduces oxidative stress → decreases Aβ deposition, Tau phosphorylation, neuronal damage, and cognitive impairment	[58]	2026
18	Animal study	C57BL/6 J male mice and 3 × Tg‐AD mice	*Lactobacillus plantarum PS128*	*Firmicutes*	Protective	Regulates propionic acid levels and GSK3β activity; reduces Aβ deposition, BACE1 expression, Tau phosphorylation, gliosis, and neuronal loss; improves cognitive function	[59]	2021
19	Animal study	*Drosophila melanogaster*	*Enterobacteria*	*Proteobacteria*	Detrimental	Immune hemocyte recruitment to the brain, triggering TNF‐JNK mediated neurodegeneration and oxidative stress	[64]	2017
20	Animal study	5xFAD mouse	*Escherichia coli*	*Proteobacteria*	Detrimental	*E. coli* colonization → MHCII antigen presentation in brain myeloid cells → promotes CD8+ T cell infiltration, suppresses the transition of microglia to a DAM, increases insoluble Aβ deposition → exacerbated cognitive decline	[65]	2025
21	Animal study	Wild‐type C57BL/6J, App^NL‐G‐F^ mice	*Helicobacter pylori* (gastric pathogen)	*Proteobacteria*	Detrimental	*H. pylori* OMVs cross biological barriers and reach the brain; taken up by astrocytes → C3‐C3aR signaling activation → glial cell activation and neuronal dysfunction → exacerbates Aβ pathology and cognitive decline	[69]	2023
22	Cell experiment	AGS cells; LN229 astrocytoma cells; neuronal and neuron‐astrocyte co‐culture	*Helicobacter pylori* (conditioned media from clinical isolates)	*Proteobacteria*	Detrimental	*H. pylori* conditioned media activates STAT1/STAT3 signaling → APP and APOE4 expression↑ → ROS↑ → astrogliosis (GFAP↑); STAT3 inhibition reduces pSTAT3 and AD marker expression	[66]	2024
23	Animal study	C57BL/6J and APP/PS1 mice	*Escherichia coli Nissle 1917*	*Proteobacteria*	Protective	*EcN* OMVs containing OmpA translocate to brain → suppress glial hyperactivation, reduce neuroinflammation, restore synaptic function (dendritic spine density↑), increase neuronal survival → reduce Aβ deposition and cognitive deficits; effects mediated via humoral (circulatory) and vagal pathways	[72]	2026
24	Animal study	C57BL/6 mice	*Escherichia fergusonii* (isolated from MCI patients)	*Proteobacteria*	Detrimental	MCI‐derived *E. fergusonii* induces TNF‐α expression in intestinal epithelial cells → LPS/TLR4/NF‐κB‐mediated neuroinflammation → cognitive impairment	[67]	2024
25	Animal study	3xTg‐AD Transgenic Mice	*Klebsiella pneumoniae*	*Proteobacteria*	Detrimental	Antibiotic‐induced dysbiosis promotes *K. pneumoniae* translocation across gut epithelial barrier → bloodstream → crosses BBB → brain infection → neuroinflammation → total tau↑ in brain → neurobehavioral impairment	[68]	2024

*Note:* This table summarizes the effects of specific gut microbial taxa on AD pathology and cognitive function, as reported in experimental studies.

Abbreviations: 12‐HHTrE, 12‐hydroxyheptadecatrienoic acid; ACSL4, acyl‐CoA synthetase long‐chain family member 4; AEP, asparagine endopeptidase; AhR, aryl hydrocarbon receptor; Akt, protein kinase B; APOE4, apolipoprotein E4; APP, amyloid precursor protein; Aβ, amyloid‐beta; BACE1, beta‐site APP cleaving enzyme 1; Bax, Bcl‐2‐associated X protein; BBB, blood–brain barrier; Bcl‐2, B‐cell lymphoma 2; BDNF, brain‐derived neurotrophic factor; C/EBPβ, CCAAT/enhancer‐binding protein beta; C3‐C3aR, complement component 3–C3a receptor; CB2, cannabinoid receptor type 2; CD, cluster of differentiation; CDK5, cyclin‐dependent kinase 5; CREB, cAMP response element‐binding protein; DAM, disease‐associated microglia; DC, dendritic cell; EPS, exopolysaccharide; FMT, fecal microbiota transplantation; GABA, gamma‐aminobutyric acid; GFAP, glial fibrillary acidic protein; GPR119, G protein‐coupled receptor 119; GPR81, G protein‐coupled receptor 81; GPX4, glutathione peroxidase 4; GSK‐3β, glycogen synthase kinase 3 beta; HIF1α, hypoxia‐inducible factor 1‐alpha; HO‐1, heme oxygenase 1; IL, interleukin; ILA, indole‐3‐lactic acid; IPA, indole‐3‐propionic acid; JAK/STAT3, Janus kinase/signal transducer and activator of transcription 3; JNK, c‐Jun N‐terminal kinase; LPC, lysophosphatidylcholine; MHCII, major histocompatibility complex class II; MYD88, myeloid differentiation primary response 88; NF‐κB, nuclear factor kappa B; NLRP3, NLR family pyrin domain containing 3; NRF2, nuclear factor erythroid 2‐related factor 2; OmpA, outer membrane protein A; OMV, outer membrane vesicle; PC, phosphatidylcholine; PGE2, prostaglandin E2; PI3K, phosphoinositide 3‐kinase; PLA2, phospholipase A2; p‐Tau, phosphorylated tau; PUFA, polyunsaturated fatty acid; ROS, reactive oxygen species; SAA, serum amyloid A; SCFA, short‐chain fatty acid; SOD2, superoxide dismutase 2; STAT, signal transducer and activator of transcription; Th1, T helper 1; TLR4, Toll‐like receptor 4; TMAO, trimethylamine N‐oxide; TNF, tumor necrosis factor.

**FIGURE 1:**
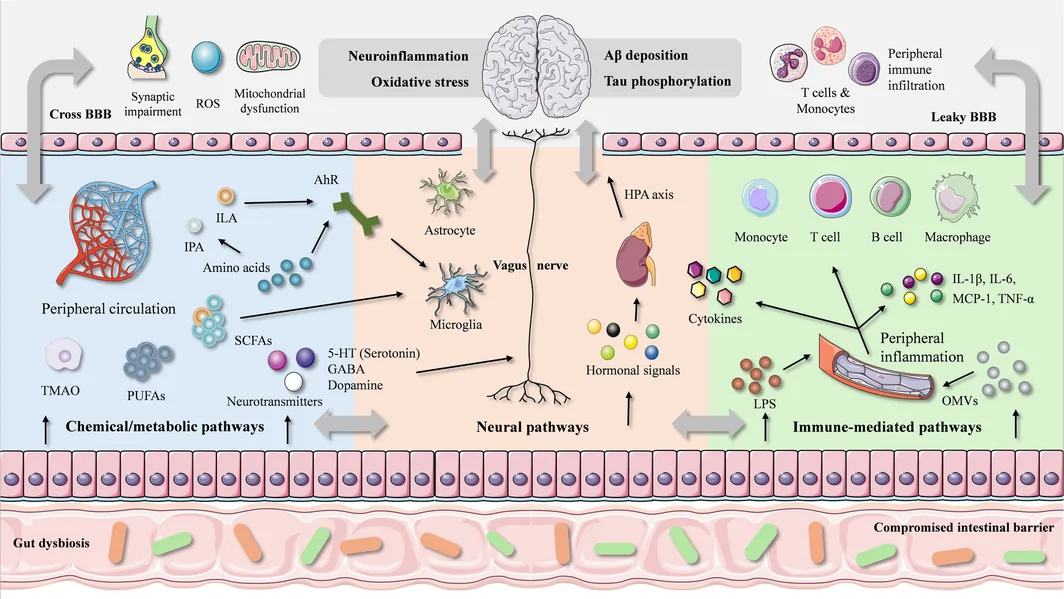
Schematic illustration of gut‐brain signaling pathways in AD pathogenesis. Gut dysbiosis compromises intestinal barrier integrity, allowing microbial signals to reach the brain through three interconnected axes. Chemical/metabolic pathways involve microbial metabolites and neurotransmitters that enter the peripheral circulation, cross the BBB, and modulate neurodegenerative processes via specific receptors. Immune‐mediated pathways are driven by microbial structural components that activate peripheral immune cells and trigger systemic inflammation, promoting peripheral immune infiltration and microglial activation. Neural pathways operate through the vagus nerve and the HPA axis. Collectively, these three axes converge to drive neuroinflammation, Aβ deposition, tau phosphorylation, oxidative stress, mitochondrial dysfunction, and synaptic impairment, culminating in AD pathology. 5‐HT, 5‐hydroxytryptamine; AD, Alzheimer's disease; AhR, aryl hydrocarbon receptor; Aβ, amyloid‐β; BBB, blood–brain barrier; GABA, gamma‐aminobutyric acid; HPA axis, hypothalamic–pituitary–adrenal axis; IL, interleukin; LPS, lipopolysaccharide; MCP‐1, monocyte chemoattractant protein‐1; OMVs, outer membrane vesicles; PUFAs, polyunsaturated fatty acids; ROS, reactive oxygen species; SCFAs, short‐chain fatty acids; TMAO, trimethylamine N‐oxide; TNF‐α, tumor necrosis factor‐alpha.

Based on the routes through which these signals reach the central nervous system, gut‐brain communication can be broadly divided into three interconnected axes: chemical/metabolic pathways, immune‐mediated pathways, and neural pathways (Figure 1).

#### 3.5.1 Chemical/Metabolic Pathways

Microbial metabolites represent the most direct signaling arm of the gut‐brain axis. SCFAs, tryptophan derivatives (including IPA and ILA), and other bioactive molecules enter the portal circulation and may reach the brain directly or influence host metabolism at peripheral sites [102, 103]. The gut microbiota also influences neurotransmitter systems, modulating the production of serotonin, GABA, and dopamine that can affect brain function through the gut‐brain axis [104]. Metabolites exert divergent effects. Some support microglial homeostasis, maintain BBB integrity, and suppress neuroinflammation through receptors such as AhR [105]. Others, including TMAO and certain PUFA metabolites, promote oxidative stress, tau phosphorylation, or inflammatory cascades [38, 61]. The net effect on the brain depends on the compositional and functional configuration of the microbial community.

#### 3.5.2 Immune‐Mediated Pathways

Along with the bioactive molecules mentioned above, structural components such as LPS and OMVs activate peripheral immune cells upon entering the circulation, triggering systemic inflammation [67, 69]. This peripheral inflammation can compromise BBB integrity, facilitating the entry of inflammatory mediators and immune cells into the brain [106]. Within the central nervous system, microglial activation and peripheral immune infiltration amplify neuroinflammation. Conversely, certain microbial signals can also suppress inflammatory responses and maintain immune homeostasis, indicating that the immune axis is bidirectional.

#### 3.5.3 Neural Pathways

The vagus nerve serves as a direct anatomical conduit between the gut and the brain [107, 108]. Vagal afferent fibers in the intestinal mucosa can detect chemical and mechanical signals from the gastrointestinal tract, and their activity is modulated by various hormonal signals [109]. These signals are transmitted to the brainstem and then to higher brain regions. Beyond the vagus nerve, neuroendocrine regulation also contributes to gut‐brain communication. The hypothalamic–pituitary–adrenal (HPA) axis integrates stress and metabolic signals, and its dysregulation has been linked to neuroinflammation in AD [110]. The vagus nerve can also mediate anti‐inflammatory effects through the cholinergic reflex, providing a rapid communication route that complements slower humoral and metabolic signaling [111].

#### 3.5.4 Integration and Crosstalk

The three signaling axes do not operate in isolation, but form a highly integrated network [112]. Barrier disruption amplifies immune activation. Metabolic signals can modulate neural excitability. Neuroinflammation can in turn compromise both intestinal and BBB integrity. This interconnectivity has therapeutic implications. Interventions targeting a single axis may elicit compensatory responses along the others, whereas strategies that concurrently modulate multiple pathways may offer synergistic benefits. Collectively, these findings establish the gut‐brain axis as a multidirectional signaling network through which gut dysbiosis may influence AD pathogenesis. This framework provides a mechanistic basis for the nutritional and metabolic interventions discussed in subsequent sections [113].

## 4 Single Nutrient, Metabolites, and AD

**FIGURE 2:**
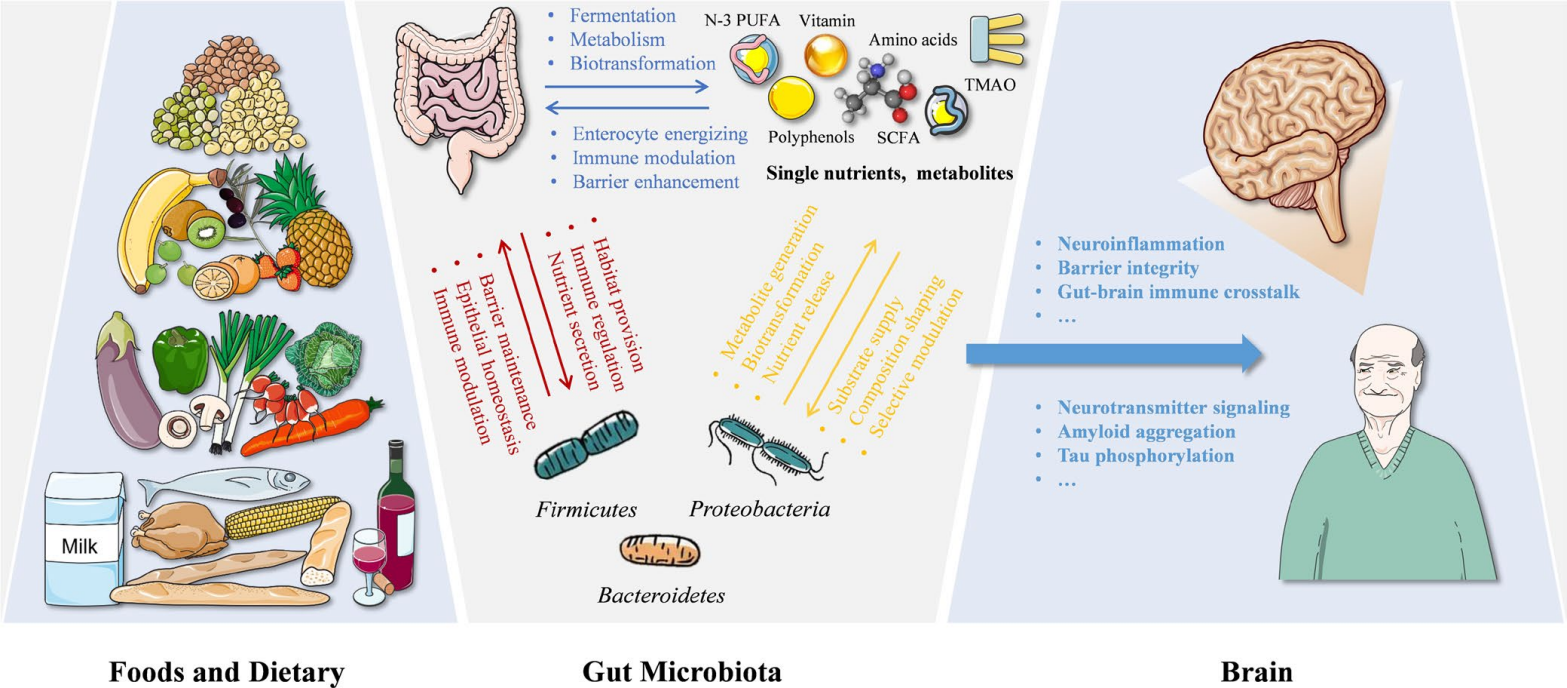
Schematic overview of the gut‐brain axis in AD. Dietary components (foods and single nutrients, e.g., vitamins, amino acids, PUFAs) modulate gut microbiota composition. Microbial metabolism, including fermentation and biotransformation, generates bioactive metabolites (e.g., SCFAs, TMAO, polyphenol derivatives) that regulate intestinal barrier function and immune responses. These gut‐derived signals influence brain pathology through neuroinflammation, barrier integrity, neurotransmitter signaling, and proteinopathy (Aβ aggregation, Tau phosphorylation).

Dietary nutrients and gut microbial metabolites represent two interconnected yet distinct categories of factors that influence AD through the gut‐brain axis. This section begins with three classes of dietary nutrients with established neuroprotective properties, namely N‐3 polyunsaturated fatty acids (N‐3 PUFA), polyphenols, and vitamins, which exert direct effects on brain health. Amino acids are then addressed as a key example of this intermediate category, functioning as both dietary constituents and metabolic substrates for gut microbiota and neurotransmitter synthesis, thereby linking nutrient intake to microbial metabolism. The section concludes with two major classes of gut microbial metabolites, short‐chain fatty acids (SCFAs) and trimethylamine‐N‐oxide (TMAO), illustrating how the gut microbiota transduces dietary components into bioactive signals that modulate AD pathogenesis. This progression from nutrients to metabolites provides a conceptual framework for understanding the continuum of diet‐microbiome‐brain interactions in AD (Figure 2).

### 4.1 N‐3 Polyunsaturated Fatty Acids

N‐3 PUFA, including eicosapentaenoic acid (EPA), docosapentaenoic acid (DPA), and docosahexaenoic acid (DHA), possess anti‐inflammatory and neuroprotective properties. A 2012 systematic review and meta‐analysis of animal studies demonstrated that long‐term N‐3 PUFA supplementation reduced Aβ burden, improved cognitive performance, and attenuated neuronal loss in AD models [114]. Recent evidence suggests that gut microbiota composition may contribute to interindividual variability in the efficacy of N‐3 PUFA supplementation [115].

Direct evidence from AD mouse models indicates that dietary N‐3 PUFA interventions modulate gut microbiota composition and inflammation. In APP/PS1 mice, EPA supplementation increased butyrate‐producing *Firmicutes*, reduced Gram‐negative *Bacteroidetes*, and concurrently downregulated MHCII^+^ microglia in the hippocampus and retina, without altering Aβ plaque burden or microglial phagocytosis [116]. Similarly, supplementation with medium‐chain triglycerides (MCTs) combined with DHA in APP/PS1 and SAMP8 mice decreased the relative abundance of *Proteobacteria* and the *Firmicutes/Bacteroidetes* ratio, accompanied by reduced serum pro‐inflammatory cytokines (IL‐2, IL‐6, MCP‐1, TNF‐α) and elevated anti‐inflammatory IL‐10 [117]. In 5 × FAD mice, DHA‐enriched egg yolk lipid supplementation increased AD‐beneficial genera including *Muribaculaceae* and *Lachnospiraceae* and suppressed microglial activation‐induced neuroinflammation [118]. A high ω‐6/ω‐3 diet (low ω‐3) significantly altered gut microbial diversity and increased genera associated with AD and inflammation in APP/PS1 mice, suggesting that low ω‐3 levels may exacerbate neuropathology through gut microbiota reorganization [119]. The causal involvement of the gut microbiota was further examined through FMT. Transfer of gut microbiota from N‐3 PUFA‐fed donors into recipient diabetes‐associated cognitive dysfunction rats recapitulated the improvements in cognitive function, neuroinflammation, and synaptic protein expression [120]. Collectively, these findings indicate that N‐3 PUFA, particularly EPA and DHA, may influence AD‐related pathology through gut microbiota modulation and attenuation of neuroinflammation, highlighting the potential of dietary N‐3 PUFA supplementation as a non‐invasive strategy to support cognitive health in the context of AD.

### 4.2 Polyphenols

Polyphenols are plant‐derived compounds characterized by phenolic rings and are abundant in fruits, vegetables, and whole grains [121]. They have been shown to reduce Aβ production, prevent Aβ aggregation, and diminish oligomer cytotoxicity [122], and mechanistic studies have further demonstrated their ability to inhibit tau hyperphosphorylation and suppress tau β‐sheet formation [123]. However, translation of these preclinical findings into clinical efficacy remains challenging due to poor bioavailability and BBB permeability [122]. Beyond these direct actions on amyloid and tau pathologies, emerging evidence suggests that polyphenols may exert neuroprotective effects through modulation of the gut–brain axis [124].

In vitro studies have shown that phenolic compounds selectively promote the growth of probiotic *lactobacilli* while inhibiting pathogenic bacteria [125], and polyphenols and their metabolites have been reported to enhance intestinal barrier integrity [126]. In naturally aged mice, a blueberry‐mulberry extract improved spatial memory, reduced hippocampal neuronal loss and brain IL‐6 and TNF‐α while increasing IL‐10, and altered gut microbiota by increasing *Lactobacillus*, *Streptococcus*, and *Lactococcus* while decreasing *Blautia*, *Lachnoclostridium*, and *Roseburia* [127]. Similarly, in D‐galactose‐induced aging mice, whole fruit phenols from kumquat reduced oxidative stress, lowered hippocampal inflammatory markers and acetylcholinesterase activity, increased acetylcholine levels, modulated gut microbiota by enriching beneficial bacteria and reducing *Bacteroides* and *Escherichia‐Shigella*, and enhanced intestinal barrier function [128]. In 5xFAD and APP/PS1 transgenic mice, Triphala improved cognitive function, modulated the APP pathway, reduced inflammation, and increased *Bacteroides*, *Proteobacteria*, and *Actinobacteria* [129]. In 3xTg‐AD mice fed a high‐fat high‐sugar diet, curcumin improved spatial working memory, accompanied by enrichment of *Oscillospiraceae* and *Rikenellaceae* at the family level and *Oscillibacter*, *Alistipes*, *Pseudoflavonifractor*, *Duncaniella*, and *Flintibacter* at the genus level. Transcriptomic analysis of the hippocampus revealed that curcumin modulated pathways related to synaptic plasticity, excitatory postsynaptic potential, and mitochondrial organization [130]. The causal involvement of gut microbiota in mediating these effects was further supported by a FMT experiment, in which transfer of microbiota from *Phyllanthus emblica* polyphenol‐treated donors into recipient mice alleviated cognitive impairment and neuroinflammation [131]. Collectively, these findings suggest that dietary polyphenols may represent a viable nutritional strategy for AD prevention, acting through both direct anti‐amyloid and anti‐tau effects and indirect modulation of the gut‐brain axis.

### 4.3 Vitamins

Vitamins are essential micronutrients categorized into fat‐soluble (A, D, E, K) and water‐soluble (B‐complex, C) groups. Epidemiological evidence has linked inadequate vitamin status to cognitive decline and increased risk of AD. Low serum vitamin D is associated with poorer neuroimaging outcomes and incident dementia [132]. This association has been confirmed by a meta‐analysis of prospective studies [133] and also supported by Mendelian randomization evidence for a causal role [134]. Vitamin E has been implicated in AD through its antioxidative and anti‐inflammatory properties [135], and emerging evidence also suggests that gut microbiota modulation may represent a potential mechanism for delaying AD progression [136].

The gut microbiota plays a bidirectional role in vitamin metabolism. Gut bacteria can synthesize certain vitamins, while vitamins in turn influence microbial composition and function [137]. Vitamin A, via its active metabolite all‐trans retinoic acid (ATRA), maintains intestinal epithelial integrity by upregulating tight junction proteins [138]. In APP/PS1 mice, vitamin A deficiency increased intestinal permeability, elevated inflammatory cytokines, exacerbated Aβ pathology, and induced gut microbiota dysbiosis and cognitive deficits, while vitamin A supplementation attenuated these pathological changes [139, 140].

Folate and vitamin B12 deficiency link to increased risk of cognitive impairment [141]. In Aβ‐infused rats, combined deficiency additively impaired memory function and disturbed gut microbiota composition [142]. Interestingly, univariate Mendelian randomization found no significant direct associations between vitamin B supplementation and AD risk. However, mediation analysis revealed that *Lachnospiraceae*, *Defluviitaleaceae*, and *Bifidobacterium* fully mediated the relationships between B12, B6, and B complex supplementation, respectively, and AD risk [91], highlighting the potential role of gut microbiota in mediating the effects of B vitamins on AD risk.

Vitamin C mitigated cognitive impairment in D‐galactose‐induced aging mice, accompanied by increased microbial diversity, particularly the enrichment of SCFA‐producing genera [143]. Collectively, these findings indicate that vitamins may influence AD pathogenesis through gut microbiota modulation, oxidative stress reduction, and systemic inflammation, underscoring the potential for personalized nutritional strategies targeting the gut‐brain axis.

### 4.4 Amino Acids and Their Metabolites

Amino acids are the building blocks of proteins and serve as precursors for neurotransmitters, microbial metabolites, and energy metabolism. A prospective cohort study suggested that lower circulating levels of essential amino acids, particularly branched‐chain amino acids, are associated with a reduced risk of AD [144]. Animal studies have further shown that dietary supplementation with essential amino acids can attenuate neuropathology and inflammation in AD‐related models [145, 146]. Beyond serving as direct nutrients, amino acids also function as key metabolic mediators linking gut microbiota dysbiosis to AD pathology. During AD progression, gut microbiota dysbiosis causes an abnormal elevation of phenylalanine and isoleucine, which promote Th1 cell infiltration into the brain, contributing to M1 microglial activation and neuroinflammation [147].

Among amino acids, tryptophan metabolism is a central pathway in microbiota‐gut‐brain communication. Tryptophan is metabolized along three principal routes: the kynurenine pathway, the serotonin pathway, and the indole pathway [148]. The kynurenine pathway produces metabolites such as kynurenine and quinolinic acid that are implicated in neuroinflammation and excitotoxicity. In parallel, the indole pathway generates metabolites that activate the aryl hydrocarbon receptor (AhR), a ligand‐activated transcription factor enriched in brain microvessels [149]. Of note, AhR activation exerts context‐dependent effects in AD. On one hand, AhR signaling has been associated with BBB disruption and neuroinflammation [150]. On the other, AhR activation by indole‐3‐acetic acid (IAA) has been linked to neuroprotective outcomes, with IAA levels positively correlating with cognitive performance. In APP/PS1 mice, *Akkermansia muciniphila* administration increased serum IAA and tryptophan levels and activated the AhR/NF‐κB/NLRP3 pathway, reducing Aβ deposition and neuroinflammation [151].

Other amino acids have also been implicated in AD through microbiota‐dependent mechanisms. In a white matter injury model, reduced L‐arginine was associated with cognitive decline. *Lachnospiraceae* were identified as a primary source of gut‐derived L‐arginine, and supplementation with either L‐arginine or *Lachnospiraceae* alleviated the decline [152]. Dietary methionine restriction improved cognition in APP/PS1 mice through gut microbiota‐dependent mechanisms and inflammation‐related mechanisms, involving modulation of tryptophan metabolism [153], SCFA signaling [154], and suppression of astrocyte‐mediated neuroinflammation via the TGF‐β1/CCN2/NF‐κB pathway [155]. Furthermore, high methionine intake was reported to impair cognition in non‐transgenic mice [156]. Isoleucine restriction also improved cognitive function in high‐fat diet‐induced obese mice through the gut‐brain axis [157].

Collectively, these findings indicate that amino acids act as both essential nutrients and metabolic intermediates through which gut microbiota influences AD pathogenesis, suggesting that targeting amino acid metabolism through dietary or microbiota‐directed strategies may offer a viable approach for gut‐brain axis‐based intervention.

### 4.5 Short‐Chain Fatty Acids

SCFAs, primarily including acetate, propionate, butyrate, isobutyrate, valerate, and isovalerate, are produced by gut microbiota through dietary fiber fermentation [158]. Multiple lines of evidence have observed alterations in gut microbial composition and SCFA profiles in AD patients and at‐risk populations. For instance, SCFA‐producing families *Lachnospiraceae* and *Bacteroidaceae*, genus *Ruminococcus* (phylum *Firmicutes*) were significantly reduced in AD patients [45]. Similarly, an *Anaerostipes*‐enriched enterosignature (ES‐Ana) positively associated with butyrate biosynthesis decreased progressively from healthy controls to AD patients and correlated positively with cognitive scores [159]. In cognitively unimpaired adults, higher cerebral Aβ burden was associated with distinct gut microbial and SCFA profiles, suggesting that gut dysbiosis and SCFA alterations occur before symptom onset [160]. Additionally, fecal propionate, isovalerate, and propionate‐producing bacteria were inversely associated with amyloid status, with propionate mediating the link between gut microbes and AD biomarkers [161].

The protective effects of SCFAs against AD are supported by multiple mechanistic studies. In AD mouse models, butyrate increases hippocampal histone acetylation levels and upregulates the expression of genes related to associative learning, thereby restoring memory function [162]. Valerate, butyrate, and propionate disrupt toxic soluble Aβ aggregation [163]. Propionate and butyrate can inhibit LPS‐induced cytokines IL‐6 and IL‐12p40 expression in human mature dendritic cells [164], suggesting that SCFAs may attenuate neuroinflammation [165]. Of note, while butyrate exerts consistent protective effects against AD, propionate may be either beneficial or detrimental depending on the specific pathway involved [166].

Beyond direct effects, SCFAs also influence central nervous system function indirectly through the gut‐brain axis [167]. In the gut, they modulate intestinal pH [168], promote mucin secretion [169], regulate intestinal permeability [170], enhance gut immune barrier function [171], provide energy for colonocytes [172], and increase mineral absorption [173]. These local actions, together with direct effects on BBB integrity and glial cells, as well as modulation of immune and vagal pathways, enable SCFAs to impact neuronal homeostasis and AD pathogenesis [174, 175, 176].

Functional analysis further revealed that higher Aβ burden correlated with a shift from anabolic biosynthesis (synthesis of beneficial metabolites, e.g., L‐arginine biosynthesis) to fermentative pathways (a catabolic state linked to dysbiosis, e.g., pyruvate fermentation to acetone). This finding implies that restoring overall microbial functional homeostasis may be more critical than simply replenishing SCFA‐producing bacteria, supporting early intervention before symptom onset as a promising strategy to modify AD progression [177].

### 4.6 Trimethylamine‐N‐Oxide

TMAO is a gut microbiota‐derived metabolite produced from dietary choline, betaine, and L‐carnitine [178], with final oxidation in the liver primarily by flavin monooxygenase 3 (FMO3) [179]. Dietary patterns influence gut‐derived TMAO levels: plant‐based diets associated with lower production [180], whereas long‐term high‐fat diets alter intestinal epithelial physiology, increase *Enterobacteriaceae* abundance, and enhance choline catabolism by *Escherichia coli* , thereby elevating circulating TMAO [181].

Multiple observational studies consistently report elevated TMAO levels in AD patients. Vogt et al. found that cerebrospinal fluid (CSF) TMAO levels were significantly higher in AD patients and correlated with biomarkers of AD pathology and neuronal degeneration [182]. Meta‐analyses have further confirmed the positive association between TMAO and cognitive impairment [183, 184]. Mechanistically, TMAO may induce cognitive dysfunction and brain aging via inhibition of the mTOR signaling pathway, leading to impaired synaptic plasticity [185]. A computational pathway analysis identified TMAO as the top‐ranked metabolite associated with AD biomarkers, revealing nine shared genetic pathways underlying both AD and TMAO metabolism [186].

Recent studies provide direct causal evidence linking specific gut microbes to AD via TMAO. Lv et al. found that *Blautia coccoides* (a species of *Bacillota*) abundance is increased in AD patients and predicts p‐Tau181 levels; supplementation with this bacterium exacerbated cognitive deficits and Tau pathology in AD mice via TMAO‐HIF1α signaling [61]. Zhang et al. showed that cognitive training reduced TMAO levels in AD patients, correlating with changes in *Roseburia*, *Lactobacillus*, and *Ruminococcus*; in APP/PS1 mice, TMAO supplementation impaired cognition, disrupted BBB, and increased Aβ deposition [187].

However, a bidirectional MR study found no significant causal association between genetically predicted TMAO and AD risk, nor for its precursor choline, betaine, or L‐carnitine; reverse analysis also found no causal effect of AD on TMAO levels [188]. Interestingly, another study found that TMAO may enhance BBB integrity and protect against inflammatory damage, whereas its precursor trimethylamine (TMA) exerts opposite effects [189]. These conflicting findings suggest that the role of TMAO in AD is complex and context‐dependent, warranting further investigation. Future studies integrating large population cohorts and mechanistic research are needed to clarify its exact role.

## 5 Dietary Patterns and AD

Diet is increasingly recognized as a comprehensive concept comprised of nutrients, foods, trace elements, macronutrients, and their complex interactions [190]. While most research on nutrition and AD risk has focused on individual nutrients, it often overlooks the complex interplay, the synergies and antagonisms, among different dietary components that fundamentally shape human diets [191]. Unhealthy dietary patterns, such as the Western diet, exemplify how such interactions can produce detrimental health outcomes. Studies show that Western diet‐induced metabolic syndrome initiates a pathogenic cascade from obesity, adipose tissue inflammation, and gut dysbiosis to exacerbated systemic inflammation, culminating in BBB damage, neuroinflammation, toxic amyloid accumulation, and finally, synaptic dysfunction, neurodegeneration, and cognitive impairment [192, 193]. Numerous animal and human studies have confirmed that high‐fat diets cause alterations in gut microbiota composition and diversity, such as a 50% reduction in bacterial abundance and an increased ratio of *Firmicutes* to *Bacteroidetes* [194, 195]. Moreover, recent evidence in mice fed an atherogenic high‐fat diet revealed that live bacteria can translocate from the gut to the brain via the vagus nerve, a phenomenon reversible upon return to normal diet [196]. Similarly, a fiber‐deficient diet, mimicking another feature of Western dietary patterns, induced cognitive impairment in mice, accompanied by hippocampal synaptic damage, microglial‐mediated synaptic engulfment, and reduced SCFA production. Gut microbiota changes were detectable as early as 7 days before cognitive decline [197]. Collectively, metabolic syndrome and gut microbiota dysbiosis, resulting from unhealthy dietary habits, are key drivers in the development of AD.

Healthy dietary patterns play a positive role in preventing AD. Studies have found that the Mediterranean diet (MeDi), the Dietary Approaches to Stop Hypertension (DASH) diet, and the Mediterranean‐DASH Intervention for Neurodegenerative Delay (MIND) diet are associated with improved cognitive scores in individuals aged 40 and over [198, 199, 200]. The MeDi is characterized by high consumption of fruits, vegetables, legumes, and fish, includes healthy fats such as olive oil, and involves moderate intake of dairy products, meat, and saturated fatty acids [201]. The neuroprotective effects of the MeDi have been mechanistically linked to its polyphenol components, particularly resveratrol and oleuropein, which modulate oxidative stress, neuroinflammation, mitochondrial function, and the gut‐brain axis through pathways such as SIRT1 activation [202]. Long‐term MeDi intervention has been reported to drive beneficial gut microbiome alterations in non‐human primates, leading to the hypothesis that positive modulation of microbial diversity, composition and function acts as a key factor mediating the health benefits of the MeDi for the host [203]. Similar to the MeDi, the DASH diet also prescribes a high intake of plant‐based foods and additionally restricts the consumption of saturated fat and total fat [204]. The MIND diet integrates principles from the MeDi and DASH diets. It emphasizes intake of natural plant‐based foods, berries, and leafy greens, restricts animal‐based and high‐saturated‐fat foods, and this dietary pattern has been demonstrated to offer neuroprotective benefits [205].

In addition to these well‐established dietary patterns, the ketogenic diet (KD) has emerged as another potential strategy for neuroprotection in AD. In a transgenic AD mouse model, a modified Mediterranean‐KD promoted *Lactobacillus* growth and lactate production, which upregulated serum metabolites that attenuated neuroinflammatory pathways in the hippocampus [206]. In a human crossover trial involving prediabetic adults with MCI, a similar diet modulated the gut microbiome and metabolome in a cognitive status‐dependent manner, including reduced GABA‐producing *Alistipes* and increased *Akkermansia muciniphila* [207]. However, the effects of KD may depend on additional environmental factors. In a mouse model combining KD with intermittent hypoxia, gut microbiota alterations, particularly *Bilophila* expansion, disrupt hippocampal function and cognitive behavior via proinflammatory immune activation. Notably, microbiome depletion prevents this cognitive decline, underscoring the causal role of gut microbes in this context [208].

Intermittent fasting (IF), which primarily includes dietary rhythm interventions such as alternate‐day fasting (ADF), time‐restricted feeding (TRF), and periodic fasting (PF), has become a major focus in nutritional research for its potential to improve brain homeostasis [209]. Studies found that IF can promote the enrichment of probiotics like *Lactobacillus*, modulate gut microbiota composition and metabolite profiles, protect intestinal barrier integrity, inhibit cerebral Aβ deposition and neuroinflammation, improve synaptic structure, and ameliorate AD‐related cognitive function [210, 211, 212, 213, 214]. In summary, promoting healthy dietary patterns and dietary rhythms holds significant clinical importance for preventing or delaying the chronic, insidious progression of AD‐related cognitive impairment.

## 6 Future Perspectives and Challenges

### 6.1 Mendelian Randomization for Causal Inference

Although MR studies have provided genetic evidence for a causal role of gut microbiota in AD (Section 3.4; Table 1), the direction of causal effects for certain taxa remains inconsistent across studies [95, 96]. These discrepancies may arise from differences in taxonomic resolution, GWAS data sources, AD outcome definitions (e.g., clinically diagnosed AD versus proxy cases, or cognitive performance metrics), and analytical methods [80, 215]. Future MR studies would benefit from standardized analytical pipelines, multi‐ancestry GWAS data, and more rigorous sensitivity analyses. Triangulating MR evidence with randomized controlled trials (RCTs) or animal experiments will also be essential to firmly establish causality.

Beyond establishing causality, recent MR studies have directly demonstrated the existence of specific microbiota‐brain structure axes causally involved in AD mechanisms (e.g., *Butyrivibrio*‐brain stem‐AD) [80]. These findings provide genetic evidence supporting the gut‐brain axis as a mechanistic framework for AD, reinforcing the rationale for microbiome‐targeted nutritional interventions. Furthermore, mediation MR analyses have identified several blood metabolites (e.g., glutamine, alanine, indole‐3‐carboxylate, and other amino acid derivatives) [78, 81, 82], immune cell phenotypes (e.g., effector memory CD4^+^ T cells, CD45 on Mo MDSC) [83, 92], lipid‐related mediators [88], vitamin‐related pathways [91], hub genes [79, 94], and epigenetic modifiers [84] as potential mediators. Upstream nutritional factors, such as the trace elements copper and carotene, may also influence AD risk through gut microbiota mediation [216]. Collectively, these findings identify a set of gut microbiota‐related mediators that may serve both as biomarkers and as direct nutritional targets for AD intervention.

Future research should therefore focus on: (1) moving beyond genus‐level associations to strain‐level and pathway‐specific causal inference using metagenomic GWAS; (2) integrating multi‐omics data (metabolomics, proteomics, immunomics, epigenomics) for network mediation analysis to identify additional mediators; (3) validating the functional relevance of MR‐identified mediators through in vitro and in vivo experiments; (4) translating these findings into precision nutrition strategies by using genetically supported mediators as biomarkers for patient stratification or as direct nutritional targets; and (5) exploring other trace elements and dietary components as upstream modulators of the gut microbiota‐AD axis.

### 6.2 From Dietary Patterns to Precision Nutrition

Dietary patterns such as the Medi, MIND, and KD exert neuroprotective effects at the population level [217]. However, individual responses vary considerably, influenced by genetic factors, gut microbiome composition, and metabolic phenotype [218]. The ApoE4 allele, the most well‐studied genetic risk factor for late‐onset AD, exemplifies this heterogeneity. ApoE4 carriers exhibit distinct alterations in microglial function, astrocyte lipid metabolism, BBB integrity, and insulin sensitivity, all of which may modulate their response to dietary interventions [219]. Preclinical evidence directly supports this notion. In APOE4‐targeted replacement mice, a KD induced genotype‐ and sex‐dependent gut microbiome and brain metabolic responses. The most pronounced benefits were observed in female APOE4 mice, whereas APOE3 mice exhibited only limited brain metabolic changes despite comparable microbiome alterations [220]. Theoretically, precision nutrition targeting metabolic pathways altered by ApoE4 could provide a tool for disease prevention. However, whether these preclinical findings translate to humans remains unclear, as long‐term clinical evidence specifically for ApoE4 carriers remains inconclusive [219]. Beyond genetic determinants, physiological responses also depend on sex, age, dietary habits, and baseline microbiome composition [221].

Precision nutrition is increasingly supported by multi‐omics technologies such as nutrigenomics, metabolomics, transcriptomics, and proteomics, which provide system‐level insights into individual metabolic responses [222]. Artificial intelligence (AI) further integrates multimodal data, including wearable biosensors, dietary tracking, and machine learning models, to generate personalized dietary recommendations [223, 224]. One ongoing clinical trial is using AI and network analysis to design personalized supplements for AD patients based on their microbiota, clinical, and dietary data, targeting disease‐associated microbial taxa and metabolic pathways [221].

Despite its promise, precision nutrition for AD prevention faces substantial hurdles. First, RCTs have failed to consistently support the protective effects observed in observational studies [225]. Second, integrating multi‐omics data and predicting individual responses remain major translational barriers [218], while AI‐driven tools face additional challenges in validation, generalizability, and ethical implementation [223]. Third, practical constraints complicate implementation. AD patients often struggle with dietary adherence, placing the burden on informal caregivers [226]. Finally, regulatory frameworks remain fragmented, as microbiome‐based products are classified variably as foods, supplements, or drugs across jurisdictions, creating inconsistencies in approval, labeling, and safety requirements [227].

Collectively, these findings suggest that transitioning from population‐level to genotype‐ and microbiome‐informed precision nutrition is a logical next step in AD prevention research. However, this transition requires rigorous validation through longitudinal human studies, standardized multi‐omics methodologies, accounting for individual variability [218, 223]. Microbiome‐informed precision medicine holds potential, yet substantial gaps remain in understanding gut microbiota‐immune‐brain interactions [228]. Addressing these gaps will require biomarker discovery, large‐scale trials, and clinical practice frameworks.

### 6.3 FMT and Next‐Generation Microbiome‐Based Therapies

Fecal microbiota transplantation (FMT) is a therapeutic procedure that transfers fecal microbiota from healthy donors to recipients. Animal studies have established FMT as a proof‐of‐concept intervention for AD. FMT remodels gut microbial composition, restores SCFAs levels, reduces neuroinflammation, and attenuates Aβ and tau pathologies, leading to cognitive improvement in transgenic mouse models [229, 230]. Mechanistically, FMT suppresses the TREM2/SYK/NF‐κB pathway in APP/PS1 mice, reducing hippocampal neuroinflammation and synaptic protein loss and reversing M1‐associated microglial polarization [231].

The efficacy of FMT depends on donor characteristics, administration protocol, and host factors. In 5xFAD mice, transplantation of cecal material from older donors increased plaque burden in the prefrontal cortex and dentate gyrus compared with material from young donors [232]. FMT from a human donor carrying the protective APOE2 allele improved short‐term recognition memory in 3xTgAD mice compared with FMT from an AD patient donor [233]. Dosage and frequency of transplantation also critically determine FMT efficacy. Every‐other‐day FMT intervention significantly improved AD pathology and symptoms, while weekly administrations failed to produce comparable improvements and cognitive benefits. Moreover, the protective effects of FMT on cognitive function were not sustained beyond 4 weeks post‐FMT cessation, elucidating the time‐effectiveness characteristics of FMT [234]. In a traumatic brain injury model, females showed less microglial suppression and distinct microglial remodeling in response to FMT compared with males, which may be attributed to sex‐specific differences in sex hormones, immune signaling pathways, and microbiota composition [235].

Despite the promise shown in animal studies, clinical translation of FMT for AD faces substantial hurdles. Available human evidence is restricted to a few case reports and small case series, with no large‐scale RCTs to date [236]. A pilot single‐arm trial in five patients with cognitive impairment demonstrated that FMT capsules were well tolerated with no adverse events. Cognitive scores improved or stabilized over 6 months in MCI cases, while severe dementia patients remained stable [237]. Two case reports of AD patients receiving FMT for recurrent *Clostridioides difficile* infection (CDI) noted cognitive gains, with MMSE scores improving from 20 to 26 at 2 months and to 29 at 6 months in an 82‐year‐old patient [238], and similar improvement in a 90‐year‐old patient after two FMT infusions [239]. While these observations are encouraging, clinical exploration of FMT as a dedicated treatment for AD remains limited [240].

Several factors contribute to the translational gap. First, the mechanisms linking gut microbiota to cognitive function are not fully understood [227]. Second, substantial interspecies differences in gut microbiota between rodents and humans, along with the challenges in selecting optimal transplant donors, pose significant obstacles [241]. Third, FMT carries risks including gastrointestinal adverse events, pathogen transmission, and potential long‐term consequences such as weight gain and metabolic changes [241]. Fourth, regulatory frameworks for FMT remain challenging. In the United States, FMT is regulated as an investigational drug, requiring an Investigational New Drug application for most uses, with FDA enforcement discretion limited to recurrent CDI unresponsive to standard treatments. FMT's classification as a live biotherapeutic product demands thorough characterization and compositional consistency; however, requirements are currently infeasible for complex fecal preparations [242]. Standardization of FMT protocols for AD patients, including donor screening, processing methods, quality control, and optimal dosing strategies, also remains a critical unmet need [243].

To address some limitations of conventional FMT, next‐generation microbiome‐based therapies are emerging [244]. Phage therapy offers a strategy to selectively eliminate pathogenic bacteria [245]. The gut virome changes with aging, and phage‐bacteriome‐metabolome interactions show distinct patterns linked to cognitive dysfunction in older adults [246]. An intermediate approach in terms of microbial complexity between whole‐stool FMT and single‐strain probiotics has also been explored. A defined consortium of eight dominant murine gut bacterial species restored gut microbial composition, reduced neuroinflammation, and Aβ plaque deposition in APP/PS1 mice, with effects superior to a single *Enterococcus* strain [247]. Beyond naturally occurring consortia, synthetic microbial communities (SynComs), composed of defined and reproducible bacterial strains, have emerged as reductionist models to dissect causal relationships between specific microbial taxa and neuroinflammation in AD, though their direct therapeutic application remains to be established [248]. Engineered bacteria represent another frontier [244]. A strain of engineered butyrate‐producing *Saccharomyces cerevisiae* (J17) rescued butyrate deficiency in AD mice and reduced Aβ plaque burden, microglia activation, and neuroinflammation [249]. Compared with FMT, which contains unknown or conditional pathogens, these approaches offer the advantage of defined composition and more predictable safety profiles [250]. However, these next‐generation therapies remain in early preclinical stages and require further validation of their dosage effects and long‐term safety.

## 7 Conclusions

In this review, we aim to systematically synthesize current evidence on how nutritional and metabolic factors modulate the gut‐microbiota‐brain axis and exert therapeutic effects in AD, integrating findings from observational studies, interventional trials, animal experiments, and MR analyses to bridge mechanistic insights with clinical translation.

Substantial evidence indicates that modifications in dietary and lifestyle habits can influence cognitive function, with certain nutrients contributing to the maintenance of neuronal homeostasis and the mitigation of cognitive decline. Nutritional intervention influences cognition and AD by modulating the gut microbiota, whose metabolites affect brain function via the gut‐brain axis. However, for achieving long‐term improvements in gut microbiota, a whole‐diet approach may hold greater promise than individual or combined nutrient supplementation. Overall, the gut microbiota constitutes a critical factor that must be considered in future research on the impact of diet on cognitive function. Consequently, a deeper understanding of the underlying mechanisms governing the interactions among nutrition, the microbiota, and the human body—through basic, clinical, and translational research—is essential to develop effective nutritional strategies for the comprehensive prevention and management of AD.

## Author Contributions

Conceptualization and literature review: Binyue Xu and Xuhuan Li; Literature review: Shangling Dong, Zheyuan Zhang, and Dong Jin; Figure design and visualization: Binyue Xu; Original draft preparation: Binyue Xu and Guoping Li; Review and editing: Jiesheng Wang. All authors have read and agreed to the published version of the manuscript.

## Ethics Statement

The authors have nothing to report.

## Consent

The authors have nothing to report.

## Conflicts of Interest

The authors declare no conflicts of interest.

## Data Availability

The data that support the findings of this study are openly available in PUBMED at https://pubmed.ncbi.nlm.nih.gov/.

## References

[1] S. Tiwari , V. Atluri , A. Kaushik , A. Yndart , and M. Nair , “Alzheimer's Disease: Pathogenesis, Diagnostics, and Therapeutics,” International Journal of Nanomedicine 14 (2019): 5541–5554. 31410002 10.2147/IJN.S200490 PMC6650620

[2] L. Narayanan and A. D. Murray , “What Can Imaging Tell Us About Cognitive Impairment and Dementia?,” World Journal of Radiology 8, no. 3 (2016): 240–254. 27029053 10.4329/wjr.v8.i3.240 PMC4807333

[3] B. J. Hanseeuw , R. A. Betensky , H. Jacobs , et al., “Association of Amyloid and Tau With Cognition in Preclinical Alzheimer Disease: A Longitudinal Study,” JAMA Neurology 76, no. 8 (2019): 915–924. 31157827 10.1001/jamaneurol.2019.1424 PMC6547132

[4] T. Ngandu , J. Lehtisalo , A. Solomon , et al., “A 2 Year Multidomain Intervention of Diet, Exercise, Cognitive Training, and Vascular Risk Monitoring Versus Control to Prevent Cognitive Decline in At‐Risk Elderly People (FINGER): A Randomised Controlled Trial,” Lancet 385, no. 9984 (2015): 2255–2263. 25771249 10.1016/S0140-6736(15)60461-5

[5] G. Livingston , A. Sommerlad , V. Orgeta , et al., “Dementia Prevention, Intervention, and Care,” Lancet 390, no. 10113 (2017): 2673–2734. 28735855 10.1016/S0140-6736(17)31363-6

[6] D. E. Barnes and K. Yaffe , “The Projected Effect of Risk Factor Reduction on Alzheimer's Disease Prevalence,” Lancet Neurology 10, no. 9 (2011): 819–828. 21775213 10.1016/S1474-4422(11)70072-2 PMC3647614

[7] G. Livingston , J. Huntley , A. Sommerlad , et al., “Dementia Prevention, Intervention, and Care: 2020 Report of the Lancet Commission,” Lancet 396, no. 10248 (2020): 413–446. 32738937 10.1016/S0140-6736(20)30367-6 PMC7392084

[8] D. E. Izquierdo , R. O. R. Gutiérrez , C. M. Andrés , et al., “Nutritional Status Assessment in Alzheimer Disease and Its Influence on Disease Progression,” Neurologia (English Edition) 37, no. 9 (2022): 735–747. 10.1016/j.nrleng.2019.11.006 34657824

[9] G. M. McKhann , D. S. Knopman , H. Chertkow , et al., “The Diagnosis of Dementia due to Alzheimer's Disease: Recommendations From the National Institute on Aging‐Alzheimer's Association Workgroups on Diagnostic Guidelines for Alzheimer's Disease,” Alzheimer's & Dementia 7, no. 3 (2011): 263–269. 10.1016/j.jalz.2011.03.005 PMC3312024 21514250

[10] B. Li , Y. He , J. Ma , et al., “Mild Cognitive Impairment Has Similar Alterations as Alzheimer's Disease in Gut Microbiota,” Alzheimer's & Dementia 15, no. 10 (2019): 1357–1366. 10.1016/j.jalz.2019.07.002 31434623

[11] C. Jiang , G. Li , P. Huang , Z. Liu , and B. Zhao , “The Gut Microbiota and Alzheimer's Disease,” Journal of Alzheimer's Disease 58, no. 1 (2017): 1–15. 10.3233/JAD-161141 28372330

[12] K. P. Scott , S. W. Gratz , P. O. Sheridan , H. J. Flint , and S. H. Duncan , “The Influence of Diet on the Gut Microbiota,” Pharmacological Research 69, no. 1 (2013): 52–60. 23147033 10.1016/j.phrs.2012.10.020

[13] R. P. Friedland , “Mechanisms of Molecular Mimicry Involving the Microbiota in Neurodegeneration,” Journal of Alzheimer's Disease 45, no. 2 (2015): 349–362. 10.3233/JAD-142841 25589730

[14] V. Berti , J. Murray , M. Davies , et al., “Nutrient Patterns and Brain Biomarkers of Alzheimer's Disease in Cognitively Normal Individuals,” Journal of Nutrition, Health & Aging 19, no. 4 (2015): 413–423. 10.1007/s12603-014-0534-0 PMC4375781 25809805

[15] V. Tremaroli and F. Bäckhed , “Functional Interactions Between the Gut Microbiota and Host Metabolism,” Nature 489, no. 7415 (2012): 242–249. 22972297 10.1038/nature11552

[16] A. B. Shreiner , J. Y. Kao , and V. B. Young , “The Gut Microbiome in Health and in Disease,” Current Opinion in Gastroenterology 31, no. 1 (2015): 69–75. 25394236 10.1097/MOG.0000000000000139 PMC4290017

[17] A. Cattaneo , N. Cattane , S. Galluzzi , et al., “Association of Brain Amyloidosis With Pro‐Inflammatory Gut Bacterial Taxa and Peripheral Inflammation Markers in Cognitively Impaired Elderly,” Neurobiology of Aging 49 (2017): 60–68. 27776263 10.1016/j.neurobiolaging.2016.08.019

[18] T. Harach , N. Marungruang , N. Duthilleul , et al., “Reduction of Abeta Amyloid Pathology in APPPS1 Transgenic Mice in the Absence of Gut Microbiota,” Scientific Reports 7 (2017): 41802. 28176819 10.1038/srep41802 PMC5297247

[19] R. Medzhitov , “Recognition of Microorganisms and Activation of the Immune Response,” Nature 449, no. 7164 (2007): 819–826. 17943118 10.1038/nature06246

[20] M. R. Minter , C. Zhang , V. Leone , et al., “Antibiotic‐Induced Perturbations in Gut Microbial Diversity Influences Neuro‐Inflammation and Amyloidosis in a Murine Model of Alzheimer's Disease,” Scientific Reports 6 (2016): 30028. 27443609 10.1038/srep30028 PMC4956742

[21] H. M. Wexler , “Bacteroides: The Good, the Bad, and the Nitty‐Gritty,” Clinical Microbiology Reviews 20, no. 4 (2007): 593–621. 17934076 10.1128/CMR.00008-07 PMC2176045

[22] H. Zafar and M. J. Saier , “Gut Bacteroides Species in Health and Disease,” Gut Microbes 13, no. 1 (2021): 1–20. 10.1080/19490976.2020.1848158 PMC7872030 33535896

[23] L. V. Hooper , T. Midtvedt , and J. I. Gordon , “How Host‐Microbial Interactions Shape the Nutrient Environment of the Mammalian Intestine,” Annual Review of Nutrition 22 (2002): 283–307. 10.1146/annurev.nutr.22.011602.092259 12055347

[24] M. Freitas , E. Tavan , C. Cayuela , L. Diop , C. Sapin , and G. Trugnan , “Host‐Pathogens Cross‐Talk. Indigenous Bacteria and Probiotics Also Play the Game,” Biology of the Cell 95, no. 8 (2003): 503–506. 14630386 10.1016/j.biolcel.2003.08.004

[25] S. Pareek , T. Kurakawa , B. Das , et al., “Comparison of Japanese and Indian Intestinal Microbiota Shows Diet‐Dependent Interaction Between Bacteria and Fungi,” npj Biofilms and Microbiomes 5, no. 1 (2019): 37. 31885873 10.1038/s41522-019-0110-9 PMC6925221

[26] S. N. Heinritz , E. Weiss , M. Eklund , et al., “Impact of a High‐Fat or High‐Fiber Diet on Intestinal Microbiota and Metabolic Markers in a Pig Model,” Nutrients 8, no. 5 (2016): 317. 27223303 10.3390/nu8050317 PMC4882729

[27] Z. Q. Zhuang , L. L. Shen , W. W. Li , et al., “Gut Microbiota Is Altered in Patients With Alzheimer's Disease,” Journal of Alzheimer's Disease 63, no. 4 (2018): 1337–1346. 10.3233/JAD-180176 29758946

[28] X. Liang , Y. Fu , W. T. Cao , et al., “Gut Microbiome, Cognitive Function and Brain Structure: A Multi‐Omics Integration Analysis,” Translational Neurodegeneration 11, no. 1 (2022): 49. 36376937 10.1186/s40035-022-00323-z PMC9661756

[29] M. A. Vinolo , H. G. Rodrigues , R. T. Nachbar , M. A. R. Vinolo , and R. Curi , “Regulation of Inflammation by Short Chain Fatty Acids,” Nutrients 3, no. 10 (2011): 858–876. 22254083 10.3390/nu3100858 PMC3257741

[30] J. Shimizu , T. Kubota , E. Takada , et al., “Propionate‐Producing Bacteria in the Intestine May Associate With Skewed Responses of IL10‐Producing Regulatory T Cells in Patients With Relapsing Polychondritis,” PLoS One 13, no. 9 (2018): e0203657. 30235279 10.1371/journal.pone.0203657 PMC6147427

[31] S. Mihori , F. Nichols , A. Provatas , et al., “Microbiome‐Derived Bacterial Lipids Regulate Gene Expression of Proinflammatory Pathway Inhibitors in Systemic Monocytes,” Frontiers in Immunology 15 (2024): 1415565. 38989285 10.3389/fimmu.2024.1415565 PMC11233717

[32] M. Ning , L. An , L. Dong , et al., “Causal Associations Between Gut Microbiota, Gut Microbiota‐Derived Metabolites, and Alzheimer's Disease: A Multivariable Mendelian Randomization Study,” Journal of Alzheimer's Disease 100, no. 1 (2024): 229–237. 10.3233/JAD-240082 38788075

[33] J. M. Hill , C. Clement , A. I. Pogue , S. Bhattacharjee , Y. Zhao , and W. J. Lukiw , “Pathogenic Microbes, the Microbiome, and Alzheimer's Disease (AD),” Frontiers in Aging Neuroscience 6 (2014): 127. 24982633 10.3389/fnagi.2014.00127 PMC4058571

[34] J. M. Hill and W. J. Lukiw , “Microbial‐Generated Amyloids and Alzheimer's Disease (AD),” Frontiers in Aging Neuroscience 7 (2015): 9. 25713531 10.3389/fnagi.2015.00009 PMC4322713

[35] W. J. Lukiw , “ *Bacteroides fragilis* Lipopolysaccharide and Inflammatory Signaling in Alzheimer's Disease,” Frontiers in Microbiology 7 (2016): 1544. 27725817 10.3389/fmicb.2016.01544 PMC5035737

[36] C. Wasén , L. C. Beauchamp , J. Vincentini , et al., “Bacteroidota Inhibit Microglia Clearance of Amyloid‐Beta and Promote Plaque Deposition in Alzheimer's Disease Mouse Models,” Nature Communications 15, no. 1 (2024): 3872. 10.1038/s41467-024-47683-w PMC11078963 38719797

[37] Y. Xia , Y. Xiao , Z. H. Wang , et al., “ *Bacteroides fragilis* in the Gut Microbiomes of Alzheimer's Disease Activates Microglia and Triggers Pathogenesis in Neuronal C/EBPβ Transgenic Mice,” Nature Communications 14, no. 1 (2023): 5471. 10.1038/s41467-023-41283-w PMC10482867 37673907

[38] C. Chen , J. Liao , Y. Xia , et al., “Gut Microbiota Regulate Alzheimer's Disease Pathologies and Cognitive Disorders via PUFA‐Associated Neuroinflammation,” Gut 71, no. 11 (2022): 2233–2252. 35017199 10.1136/gutjnl-2021-326269 PMC10720732

[39] E. M. Borsom , K. Conn , C. R. Keefe , et al., “Predicting Neurodegenerative Disease Using Prepathology Gut Microbiota Composition: A Longitudinal Study in Mice Modeling Alzheimer's Disease Pathologies,” Microbiology Spectrum 11, no. 2 (2023): e0345822. 36877047 10.1128/spectrum.03458-22 PMC10101110

[40] K. C. Fan , C. C. Lin , Y. L. Chiu , S. H. Koh , Y. C. Liu , and Y. F. Chuang , “Compositional and Functional Gut Microbiota Alterations in Mild Cognitive Impairment: Links to Alzheimer's Disease Pathology,” Alzheimer's Research & Therapy 17, no. 1 (2025): 122. 10.1186/s13195-025-01769-9 PMC12123878 40448221

[41] M. Azad , M. Sarker , T. Li , et al., “Probiotic Species in the Modulation of Gut Microbiota: An Overview,” BioMed Research International 2018 (2018): 9478630. 29854813 10.1155/2018/9478630 PMC5964481

[42] Y. Sun , S. Zhang , Q. Nie , et al., “Gut Firmicutes: Relationship With Dietary Fiber and Role in Host Homeostasis,” Critical Reviews in Food Science and Nutrition 63, no. 33 (2023): 12073–12088. 35822206 10.1080/10408398.2022.2098249

[43] N. Kim , M. Yun , Y. J. Oh , and H. J. Choi , “Mind‐Altering With the Gut: Modulation of the Gut‐Brain Axis With Probiotics,” Journal of Microbiology 56, no. 3 (2018): 172–182. 29492874 10.1007/s12275-018-8032-4

[44] M. Azad , M. Sarker , and D. Wan , “Immunomodulatory Effects of Probiotics on Cytokine Profiles,” BioMed Research International 2018 (2018): 8063647. 30426014 10.1155/2018/8063647 PMC6218795

[45] P. Liu , L. Wu , G. Peng , et al., “Altered Microbiomes Distinguish Alzheimer's Disease From Amnestic Mild Cognitive Impairment and Health in a Chinese Cohort,” Brain, Behavior, and Immunity 80 (2019): 633–643. 31063846 10.1016/j.bbi.2019.05.008

[46] J. P. Haran , S. K. Bhattarai , S. E. Foley , et al., “Alzheimer's Disease Microbiome Is Associated With Dysregulation of the Anti‐Inflammatory P‐Glycoprotein Pathway,” MBio 10, no. 3 (2019): e00632‐19. 31064831 10.1128/mBio.00632-19 PMC6509190

[47] C. C. Hung , C. C. Chang , C. W. Huang , R. Nouchi , and C. H. Cheng , “Gut Microbiota in Patients With Alzheimer's Disease Spectrum: A Systematic Review and Meta‐Analysis,” Aging (Albany NY) 14, no. 1 (2022): 477–496. 35027502 10.18632/aging.203826 PMC8791218

[48] A. Ueda , S. Shinkai , H. Shiroma , et al., “Identification of *Faecalibacterium prausnitzii* Strains for Gut Microbiome‐Based Intervention in Alzheimer's‐Type Dementia,” Cell Reports Medicine 2, no. 9 (2021): 100398. 34622235 10.1016/j.xcrm.2021.100398 PMC8484692

[49] L. Li , C. Yang , M. Jia , et al., “Synbiotic Therapy With *Clostridium sporogenes* and Xylan Promotes Gut‐Derived Indole‐3‐Propionic Acid and Improves Cognitive Impairments in an Alzheimer's Disease Mouse Model,” Food & Function 15, no. 15 (2024): 7865–7882. 38967039 10.1039/d4fo00886c

[50] Y. Shiqing , L. Xinjie , Z. Xiaotong , et al., “ *Clostridium butyricum* Enhances Cognitive Function in APP/PS1 Mice by Modulating Neuropathology and Regulating Acetic Acid Levels in the Gut Microbiota,” Microbiology Spectrum 13, no. 8 (2025): e0017825. 40621927 10.1128/spectrum.00178-25 PMC12323352

[51] J. L. A. Jarne‐Ferrer , C. Griñán‐Ferré , B. Jora , et al., “Soluble Epoxide Hydrolase Inhibition Improves Alzheimer's Disease Hallmarks: Correlation With Peripheral Inflammation and Gut Microbiota Modulation,” Aging and Disease 17, no. 2 (2025): 1131–1154. 40423637 10.14336/AD.2025.0201 PMC12834399

[52] C. Yang , J. Sun , L. Li , et al., “Synbiotics of *Lactobacillus suilingensis* and Inulin Alleviates Cognitive Impairment via Regulating Gut Microbiota Indole‐3‐Lactic Acid Metabolism in Female AD Mice,” Alzheimer's & Dementia 21, no. 7 (2025): e70406. 10.1002/alz.70406 PMC12202469 40572043

[53] H. Kim , E. Lee , M. Park , et al., “Microbiome‐Derived Indole‐3‐Lactic Acid Reduces Amyloidopathy Through Aryl‐Hydrocarbon Receptor Activation,” Brain, Behavior, and Immunity 122 (2024): 568–582. 39197546 10.1016/j.bbi.2024.08.051

[54] L. Qiao , Y. Chen , X. Song , X. Dou , and C. Xu , “Selenium Nanoparticles‐Enriched *Lactobacillus casei* ATCC 393 Prevents Cognitive Dysfunction in Mice Through Modulating Microbiota‐Gut‐Brain Axis,” International Journal of Nanomedicine 17 (2022): 4807–4827. 36246933 10.2147/IJN.S374024 PMC9562773

[55] Y. Wang , D. Wang , H. Lv , et al., “Modulation of the Gut Microbiota and Glycometabolism by a Probiotic to Alleviate Amyloid Accumulation and Cognitive Impairments in AD Rats,” Molecular Nutrition & Food Research 66, no. 19 (2022): e2200265. 35975737 10.1002/mnfr.202200265

[56] X. Song , Z. Zhao , Y. Zhao , et al., “ *Lactobacillus plantarum* DP189 Prevents Cognitive Dysfunction in D‐Galactose/AlCl3 Induced Mouse Model of Alzheimer's Disease via Modulating Gut Microbiota and PI3K/Akt/GSK‐3β Signaling Pathway,” Nutritional Neuroscience 25, no. 12 (2022): 2588–2600. 34755592 10.1080/1028415X.2021.1991556

[57] N. A. Ahmad Alwi , S. M. Lim , V. Mani , and K. Ramasamy , “ *Lactobacillus* Spp.‐Enhanced Memory Is Strain‐Dependent and Associated, in Part, With Amyloidogenic and Anti‐Oxidant/Oxidative Stress Interplay in Amyloid Beta Precursor Protein Transgenic Mice,” Journal of Dietary Supplements 20, no. 5 (2023): 717–734. 35876040 10.1080/19390211.2022.2103608

[58] Y. Kong , X. Lv , Y. Yang , et al., “ *Lactobacillus mucosae* Reduces Neuronal Oxidative Stress in Alzheimer's Disease via the Regulation of CB2 Signaling,” Journal of Integrative Neuroscience 25, no. 5 (2026): 48598. 42216640 10.31083/JIN48598

[59] H. J. Huang , J. L. Chen , J. F. Liao , et al., “ *Lactobacillus plantarum* PS128 Prevents Cognitive Dysfunction in Alzheimer's Disease Mice by Modulating Propionic Acid Levels, Glycogen Synthase Kinase 3 Beta Activity, and Gliosis,” BMC Complementary Medicine and Therapies 21, no. 1 (2021): 259. 34627204 10.1186/s12906-021-03426-8 PMC8502419

[60] Y. Wei , Y. Huang , S. Wang , et al., “Role of *Ruminococcus gnavus* in the Elderly With Cognitive Impairment: A Case‐Control Study and an Animal Experiment,” Journal of Alzheimer's Disease 107, no. 4 (2025): 1645–1660. 10.1177/13872877251372522 40888486

[61] X. Lv , T. Ye , X. Liao , et al., “ *Blautia coccoides* ‐Derived Metabolite Trimethylamine‐N‐Oxide Exacerbates Alzheimer's Disease Progression via Targeting HIF1α Signaling,” Gut Microbes 18, no. 1 (2026): 2605768. 41459734 10.1080/19490976.2025.2605768 PMC12758303

[62] X. Wang , Z. Xie , J. Yuan , et al., “Sodium Oligomannate Disrupts the Adherence of Rib(High) Bacteria to Gut Epithelia to Block SAA‐Triggered Th1 Inflammation in 5XFAD Transgenic Mice,” Cell Discovery 10, no. 1 (2024): 115. 39557828 10.1038/s41421-024-00725-5 PMC11573985

[63] C. Sheng , K. Yang , B. He , W. du , Y. Cai , and Y. Han , “Combination of Gut Microbiota and Plasma Amyloid‐β as a Potential Index for Identifying Preclinical Alzheimer's Disease: A Cross‐Sectional Analysis From the SILCODE Study,” Alzheimer's Research & Therapy 14, no. 1 (2022): 35. 10.1186/s13195-022-00977-x PMC8843023 35164860

[64] S. C. Wu , Z. S. Cao , K. M. Chang , and J. L. Juang , “Intestinal Microbial Dysbiosis Aggravates the Progression of Alzheimer's Disease in *Drosophila* ,” Nature Communications 8, no. 1 (2017): 24. 10.1038/s41467-017-00040-6 PMC5478647 28634323

[65] L. Blackmer‐Raynolds , L. D. Lipson , A. Kozlov , et al., “Indigenous Gut Microbes Modulate Neural Cell State and Neurodegenerative Disease Susceptibility,” Cell Systems 17, no. 2 (2026): 101481. 41638211 10.1016/j.cels.2025.101481 PMC13091097

[66] M. Kandpal , B. Baral , N. Varshney , et al., “Gut‐Brain Axis Interplay via STAT3 Pathway: Implications of *Helicobacter pylori* Derived Secretome on Inflammation and Alzheimer's Disease,” Virulence 15, no. 1 (2024): 2303853. 38197252 10.1080/21505594.2024.2303853 PMC10854367

[67] X. Ma , J. K. Kim , Y. J. Shin , et al., “Lipopolysaccharide‐Producing *Veillonella infantium* and *Escherichia fergusonii* Cause Vagus Nerve‐Mediated Cognitive Impairment in Mice,” Brain, Behavior, and Immunity 118 (2024): 136–148. 38428648 10.1016/j.bbi.2024.02.031

[68] G. Park , S. Kadyan , N. Hochuli , et al., “An Enteric Bacterial Infection Triggers Neuroinflammation and Neurobehavioral Impairment in 3xTg‐AD Transgenic Mice,” Journal of Infectious Diseases 230, no. Suppl_2 (2024): S95–S108. 39255397 10.1093/infdis/jiae165 PMC11385593

[69] J. Xie , L. Cools , G. Van Imschoot , et al., “ *Helicobacter pylori* ‐Derived Outer Membrane Vesicles Contribute to Alzheimer's Disease Pathogenesis via C3‐C3aR Signalling,” Journal of Extracellular Vesicles 12, no. 2 (2023): e12306. 36792546 10.1002/jev2.12306 PMC9931688

[70] X. Zhan , B. Stamova , L. W. Jin , C. DeCarli , B. Phinney , and F. R. Sharp , “Gram‐Negative Bacterial Molecules Associate With Alzheimer Disease Pathology,” Neurology 87, no. 22 (2016): 2324–2332. 27784770 10.1212/WNL.0000000000003391 PMC5135029

[71] Y. Zhao , L. Cong , V. Jaber , and W. J. Lukiw , “Microbiome‐Derived Lipopolysaccharide Enriched in the Perinuclear Region of Alzheimer's Disease Brain,” Frontiers in Immunology 8 (2017): 1064. 28928740 10.3389/fimmu.2017.01064 PMC5591429

[72] J. Xie , X. J. Dai , Q. Li , et al., “ *Escherichia coli* Nissle 1917 Alleviates Alzheimer's Disease in Mice Through OmpA‐Containing Outer Membrane Vesicles,” Cell Reports Medicine 7, no. 5 (2026): 102781. 42066770 10.1016/j.xcrm.2026.102781 PMC13198242

[73] A. M. Jimenez‐García , M. Villarino , and N. Arias , “A Systematic Review and Meta‐Analysis of Basal Microbiota and Cognitive Function in Alzheimer's Disease: A Potential Target for Treatment or a Contributor to Disease Progression?,” Alzheimer's & Dementia (Amsterdam, Netherlands) 16, no. 4 (2024): e70057. 10.1002/dad2.70057 PMC11672027 39734582

[74] H. Li , X. Cui , Y. Lin , F. Huang , A. Tian , and R. Zhang , “Gut Microbiota Changes in Patients With Alzheimer's Disease Spectrum Based on 16S rRNA Sequencing: A Systematic Review and Meta‐Analysis,” Frontiers in Aging Neuroscience 16 (2024): 1422350. 39175809 10.3389/fnagi.2024.1422350 PMC11338931

[75] B. A. Ference , M. V. Holmes , and G. D. Smith , “Using Mendelian Randomization to Improve the Design of Randomized Trials,” Cold Spring Harbor Perspectives in Medicine 11, no. 7 (2021): a040980. 33431510 10.1101/cshperspect.a040980 PMC8247560

[76] X. Guo , X. Zhang , P. Tang , L. Chong , and R. Li , “Integration of Genome‐Wide Association Studies (GWAS) and Microbiome Data Highlights the Impact of Sulfate‐Reducing Bacteria on Alzheimer's Disease,” Age and Ageing 52, no. 7 (2023): afad112. 37466641 10.1093/ageing/afad112

[77] C. A. Emdin , A. V. Khera , and S. Kathiresan , “Mendelian Randomization,” JAMA 318, no. 19 (2017): 1925–1926. 29164242 10.1001/jama.2017.17219

[78] H. Zeng , K. Zhou , Y. Zhuang , A. Li , B. Luo , and Y. Zhang , “Unraveling the Connection Between Gut Microbiota and Alzheimer's Disease: A Two‐Sample Mendelian Randomization Analysis,” Frontiers in Aging Neuroscience 15 (2023): 1273104. 37908561 10.3389/fnagi.2023.1273104 PMC10613649

[79] J. Y. Liu , C. Y. Tan , L. Luo , and X. Yin , “Gut Microbiota May Affect Alzheimer's Disease Through Synaptic Function Mediated by CAMs Pathway: A Study Combining Mendelian Randomization and Bioinformatics,” Journal of Alzheimer's Disease Reports 9 (2025): 25424823241310719. 10.1177/25424823241310719 PMC11864269 40034528

[80] L. Ge , L. Zhu , C. Su , and Z. Jin , “Exploring Causal Gut‐Brain Axes in Alzheimer's Disease Using Mediation Mendelian Randomization Analysis,” Journal of Alzheimer's Disease 107, no. 1 (2025): 223–232. 10.1177/13872877251360004 40665707

[81] J. Ning , S. Y. Huang , S. D. Chen , Y. R. Zhang , Y. Y. Huang , and J. T. Yu , “Investigating Casual Associations Among Gut Microbiota, Metabolites, and Neurodegenerative Diseases: A Mendelian Randomization Study,” Journal of Alzheimer's Disease 87, no. 1 (2022): 211–222. 10.3233/JAD-215411 35275534

[82] G. Chen , Y. Jin , C. Chu , Y. Zheng , Y. Chen , and X. Zhu , “Genetic Prediction of Blood Metabolites Mediating the Relationship Between Gut Microbiota and Alzheimer's Disease: A Mendelian Randomization Study,” Frontiers in Microbiology 15 (2024): 1414977. 39224217 10.3389/fmicb.2024.1414977 PMC11366617

[83] X. Y. Dong , Y. Han , T. B. Wang , Y. B. Han , and L. Liu , “Mediators Between Gut Microbiota and Alzheimer's Disease: A Mediation Mendelian Randomization Study,” Journal of Alzheimer's Disease 109, no. 2 (2026): 876–888. 10.1177/13872877251401157 41334716

[84] Y. Yan , Y. Pan , S. Lu , et al., “Gut Microbiota‐Mediated Targeting of EHMT2 in Individuals of European Ancestry: A Novel Therapeutic Approach for Alzheimer's Disease,” Journal of Alzheimer's Disease 107, no. 4 (2025): 1575–1593. 10.1177/13872877251372945 40984004

[85] Q. Zhao , A. Baranova , H. Cao , and F. Zhang , “Evaluating Causal Effects of Gut Microbiome on Alzheimer's Disease,” Journal of Prevention of Alzheimer's Disease 11, no. 6 (2024): 1843–1848. 10.14283/jpad.2024.113 39559896

[86] W. Li , B. M. Lv , Y. Quan , Q. Zhu , and H. Y. Zhang , “Associations Between Serum Mineral Nutrients, Gut Microbiota, and Risk of Neurological, Psychiatric, and Metabolic Diseases: A Comprehensive Mendelian Randomization Study,” Nutrients 16, no. 2 (2024): 244. 38257137 10.3390/nu16020244 PMC10818407

[87] X. Gao and L. Wang , “Associations of Gut Bacterial Classes Clostridia and Deltaproteobacteria With Type 2 Diabetes and Alzheimer's Disease: A Two‐Sample Mendelian Randomization Study,” Medicine (Baltimore) 105, no. 20 (2026): e48685. 42152315 10.1097/MD.0000000000048685 PMC13183148

[88] Z. Liu , L. Kuang , X. Zhou , et al., “Effect of the Plasma Lipidome, Immune Cells, Inflammatory Proteins, Gut Microbiota, and Plasma Metabolites on Alzheimer's Disease: A Two‐Sample Mendelian Randomized Study and Mediation Analysis,” Brain Research 1869 (2025): 150009. 41151745 10.1016/j.brainres.2025.150009

[89] Z. L. Ren , H. H. Zhou , C. P. Chen , H. He , D. L. Wang , and Z. Liu , “Causal Relationships Between Gut Microbiota and Dementia: A Two‐Sample, Bidirectional, Mendelian Randomization Study,” World Journal of Clinical Cases 12, no. 16 (2024): 2780–2788. 38899286 10.12998/wjcc.v12.i16.2780 PMC11185324

[90] F. Lin , X. Yang , X. Xie , et al., “The Causal Association Between the New Gut Microbiota and Alzheimer's Disease: A Mendelian Randomization Study,” Journal of Alzheimer's Disease Reports 10 (2026): 25424823261422629. 10.1177/25424823261422629 PMC13039060 41929950

[91] Y. An , Z. Cao , Y. Du , et al., “Bidirectional Two‐Sample, Two‐Step Mendelian Randomisation Study Reveals Mediating Role of Gut Microbiota Between Vitamin B Supplementation and Alzheimer's Disease,” Nutrients 16, no. 22 (2024): 3929. 39599715 10.3390/nu16223929 PMC11597120

[92] H. Zhang , Y. Wang , H. Zhao , W. Wang , and F. Han , “The Involvement of Effector Memory CD4(+) T Cells in Mediating the Impact of Genus *Oscillibacter* Gut Microbiota on Alzheimer's Disease: A Mendelian Randomization Study,” Frontiers in Aging Neuroscience 16 (2024): 1423707. 39170894 10.3389/fnagi.2024.1423707 PMC11335717

[93] A. Chen , Y. Wang , and Y. Q. Hu , “Exploring Causal Relationships Between Gut Microbiota and Alzheimer's Disease: A Bidirectional Mendelian Randomization Study,” Journal of Alzheimer's Disease Reports 8, no. 1 (2024): 1031–1040. 10.3233/ADR-240071 PMC11305842 39114549

[94] T. Ding , J. Chen , Y. Xiang , et al., “The Gut‐Brain Axis in Alzheimer's: From Microbiota Genetics to Stigmasterol's Neuroprotection Mechanism,” Degenerative Neurological and Neuromuscular Disease 16 (2026): 580890. 42077232 10.2147/DNND.S580890 PMC13135099

[95] D. Ji , W. Z. Chen , L. Zhang , et al., “Gut Microbiota, Circulating Cytokines and Dementia: A Mendelian Randomization Study,” Journal of Neuroinflammation 21, no. 1 (2024): 2. 38178103 10.1186/s12974-023-02999-0 PMC10765696

[96] Z. Y. Xiong , H. M. Li , C. S. Qiu , et al., “Investigating Causal Associations Between the Gut Microbiota and Dementia: A Mendelian Randomization Study,” Nutrients 16, no. 19 (2024): 3312. 39408279 10.3390/nu16193312 PMC11479048

[97] J. Fu , Y. Qin , L. Xiao , and X. Dai , “Causal Relationship Between Gut Microflora and Dementia: A Mendelian Randomization Study,” Frontiers in Microbiology 14 (2023): 1306048. 38287957 10.3389/fmicb.2023.1306048 PMC10822966

[98] Z. Zhuang , R. Yang , W. Wang , L. Qi , and T. Huang , “Associations Between Gut Microbiota and Alzheimer's Disease, Major Depressive Disorder, and Schizophrenia,” Journal of Neuroinflammation 17, no. 1 (2020): 288. 33008395 10.1186/s12974-020-01961-8 PMC7532639

[99] J. Li , X. Hu , X. Tao , et al., “Deconstruct the Link Between Gut Microbiota and Neurological Diseases: Application of Mendelian Randomization Analysis,” Frontiers in Cellular and Infection Microbiology 15 (2025): 1433131. 40115072 10.3389/fcimb.2025.1433131 PMC11922733

[100] V. Peddinti , M. M. Avaghade , S. U. Suthar , et al., “Gut Instincts: Unveiling the Connection Between Gut Microbiota and Alzheimer's Disease,” Clinical Nutrition ESPEN 60 (2024): 266–280. 38479921 10.1016/j.clnesp.2024.02.019

[101] X. Zha , X. Liu , M. Wei , et al., “Microbiota‐Derived Lysophosphatidylcholine Alleviates Alzheimer's Disease Pathology via Suppressing Ferroptosis,” Cell Metabolism 37, no. 1 (2025): 169–186. 39510074 10.1016/j.cmet.2024.10.006

[102] X. Ji , J. Wang , T. Lan , et al., “Gut Microbial Metabolites and the Brain‐Gut Axis in Alzheimer's Disease: A Review,” Biomolecules & Biomedicine 26, no. 2 (2025): 240–250. 40791147 10.17305/bb.2025.12921 PMC12505531

[103] Q. Cao , M. Shen , R. Li , et al., “Elucidating the Specific Mechanisms of the Gut‐Brain Axis: The Short‐Chain Fatty Acids‐Microglia Pathway,” Journal of Neuroinflammation 22, no. 1 (2025): 133. 40400035 10.1186/s12974-025-03454-y PMC12093714

[104] T. Taj , M. Kaushik , A. Islam , et al., “Microbiota‐Brain Interaction: The Role of Gut‐Derived Proteins in Addressing Various Neurological Disorders Including Parkinson's (PD) and Alzheimer's Diseases (AD),” Biomedicine & Pharmacotherapy 193 (2025): 118861. 41365116 10.1016/j.biopha.2025.118861

[105] M. Peng and H. Sun , “The Indole‐Brain Connection: Neuroimmune Mechanisms and Therapy,” Current Opinion in Immunology 98 (2026): 102708. 41385980 10.1016/j.coi.2025.102708

[106] J. C. Park , L. Chang , H. K. Kwon , and S. H. Im , “Beyond the Gut: Decoding the Gut‐Immune‐Brain Axis in Health and Disease,” Cellular & Molecular Immunology 22, no. 11 (2025): 1287–1312. 40804450 10.1038/s41423-025-01333-3 PMC12575876

[107] L. Yan , H. Li , Y. Qian , et al., “Transcutaneous Vagus Nerve Stimulation: A New Strategy for Alzheimer's Disease Intervention Through the Brain‐Gut‐Microbiota Axis?,” Frontiers in Aging Neuroscience 16 (2024): 1334887. 38476661 10.3389/fnagi.2024.1334887 PMC10927744

[108] J. Liang , X. Li , C. Mao , Z. Qin , and R. Wang , “Electro‐Microbial Interplay in the Gut–Brain Axis: An Emerging Paradigm for Gut Microbiota Modulation via Vagus Nerve Stimulation,” Current Opinion in Pharmacology 86 (2026): 102588. 41421301 10.1016/j.coph.2025.102588

[109] T. L. Powley , “Brain‐Gut Communication: Vagovagal Reflexes Interconnect the Two “Brains”,” American Journal of Physiology, Gastrointestinal and Liver Physiology 321, no. 5 (2021): G576–G587. 34643086 10.1152/ajpgi.00214.2021 PMC8616589

[110] U. Mukherjee and P. H. Reddy , “Gut‐Brain Relationship in Dementia and Alzheimer's Disease: Impact on Stress and Immunity,” Ageing Research Reviews 111 (2025): 102843. 40712930 10.1016/j.arr.2025.102843

[111] S. Breit , A. Kupferberg , G. Rogler , and G. Hasler , “Vagus Nerve as Modulator of the Brain‐Gut Axis in Psychiatric and Inflammatory Disorders,” Frontiers in Psychiatry 9 (2018): 44. 29593576 10.3389/fpsyt.2018.00044 PMC5859128

[112] C. Jiang , W. M. Nageeb , S. Batool , et al., “Unraveling the Gut Microbiota‐Brain Axis: Mechanisms, Pathophysiology, and Therapeutic Opportunities,” iScience 29, no. 6 (2026): 116224. 42305614 10.1016/j.isci.2026.116224 PMC13266037

[113] C. Chen , G. Q. Wang , D. D. Li , and F. Zhang , “Microbiota‐Gut‐Brain Axis in Neurodegenerative Diseases: Molecular Mechanisms and Therapeutic Targets,” Molecular Biomedicine 6, no. 1 (2025): 64. 40952592 10.1186/s43556-025-00307-1 PMC12436269

[114] C. R. Hooijmans , P. C. M. Pasker‐de Jong , R. B. M. de Vries , et al., “The Effects of Long‐Term Omega‐3 Fatty Acid Supplementation on Cognition and Alzheimer's Pathology in Animal Models of Alzheimer's Disease: A Systematic Review and Meta‐Analysis,” Journal of Alzheimer's Disease 28, no. 1 (2012): 191–209. 10.3233/JAD-2011-111217 22002791

[115] B. E. Kerman , W. Self , and H. N. Yassine , “Can the Gut Microbiome Inform the Effects of Omega‐3 Fatty Acid Supplementation Trials on Cognition?,” Current Opinion in Clinical Nutrition and Metabolic Care 27, no. 2 (2024): 116–124. 38170690 10.1097/MCO.0000000000001007 PMC10872319

[116] B. Altendorfer , A. Benedetti , H. Mrowetz , et al., “Omega‐3 EPA Supplementation Shapes the Gut Microbiota Composition and Reduces Major Histocompatibility Complex Class II in Aged Wild‐Type and APP/PS1 Alzheimer's Mice: A Pilot Experimental Study,” Nutrients 17, no. 7 (2025): 1108. 40218866 10.3390/nu17071108 PMC11990804

[117] Z. Wang , Y. Sun , D. Zhang , et al., “Gut Microbiota and Inflammation Analyses Reveal the Protective Effect of Medium‐Chain Triglycerides Combined With Docosahexaenoic Acid on Cognitive Function in APP/PS1 and SAMP8 Mice,” Nutrition Research 132 (2024): 27–39. 39437526 10.1016/j.nutres.2024.09.015

[118] L. Cheng , Y. Wang , Y. Ma , et al., “Daily Supplementation With Egg Yolk Lipids From Two Eggs Alleviated Cognitive Impairment in 5xFAD Mice by Restoring Neuronal and Synaptic Function and Regulating Gut Microbiota,” Food & Function 17, no. 12 (2026): 5690–5703. 42300178 10.1039/d6fo00928j

[119] L. Ordoñez‐Gutierrez and F. Wandosell , “Sex Hormones and Diets Rich in Polyunsaturated ω‐6/ω‐3 Fatty Acids Modify Microbiota Distinctly in a Mouse Model of Alzheimer's Disease,” Gut Microbiome 6 (2025): e10. 40703676 10.1017/gmb.2025.10005 PMC12284841

[120] H. Huang , T. Zhao , and W. Ma , “Omega‐3 Polyunsaturated Fatty Acids Attenuate Cognitive Impairment via the Gut‐Brain Axis in Diabetes‐Associated Cognitive Dysfunction Rats,” Brain, Behavior, and Immunity 127 (2025): 147–169. 40068791 10.1016/j.bbi.2025.03.015

[121] K. B. Pandey and S. I. Rizvi , “Plant Polyphenols as Dietary Antioxidants in Human Health and Disease,” Oxidative Medicine and Cellular Longevity 2, no. 5 (2009): 270–278. 20716914 10.4161/oxim.2.5.9498 PMC2835915

[122] R. Gaudreault and N. Mousseau , “Mitigating Alzheimer's Disease With Natural Polyphenols: A Review,” Current Alzheimer Research 16, no. 6 (2019): 529–543. 30873922 10.2174/1567205016666190315093520

[123] Q. Zheng , M. T. Kebede , M. M. Kemeh , et al., “Inhibition of the Self‐Assembly of Aβ and of Tau by Polyphenols: Mechanistic Studies,” Molecules 24, no. 12 (2019): 2316. 31234523 10.3390/molecules24122316 PMC6630797

[124] F. El Gaamouch , F. Chen , L. Ho , et al., “Benefits of Dietary Polyphenols in Alzheimer's Disease,” Frontiers in Aging Neuroscience 14 (2022): 1019942. 36583187 10.3389/fnagi.2022.1019942 PMC9792677

[125] R. Pacheco‐Ordaz , A. Wall‐Medrano , M. G. Goñi , G. Ramos‐Clamont‐Montfort , J. F. Ayala‐Zavala , and G. A. González‐Aguilar , “Effect of Phenolic Compounds on the Growth of Selected Probiotic and Pathogenic Bacteria,” Letters in Applied Microbiology 66, no. 1 (2018): 25–31. 29063625 10.1111/lam.12814

[126] S. Bernardi , C. Del B , M. Marino , et al., “Polyphenols and Intestinal Permeability: Rationale and Future Perspectives,” Journal of Agricultural and Food Chemistry 68, no. 7 (2020): 1816–1829. 31265272 10.1021/acs.jafc.9b02283

[127] H. Li , C. Xiao , F. Wang , X. Guo , Z. Zhou , and Y. Jiang , “Blueberry‐Mulberry Extract Alleviates Cognitive Impairment, Regulates Gut Metabolites, and Inhibits Inflammation in Aged Mice,” Food 12, no. 4 (2023): 860. 10.3390/foods12040860 PMC9956669 36832936

[128] F. Li , Y. Yi , J. Zhao , L. Lei , K. Zeng , and J. Ming , “Enhancing Cognitive Memory Function With Kumquat ( *Fortunella japonica* Swingle) Polyphenol Extract: Mechanisms of Action in D‐Galactose‐Induced Aging Model Mice,” Food & Function 16, no. 12 (2025): 4802–4821. 40407011 10.1039/d4fo06134a

[129] P. Upadhyay , A. Tyagi , S. Agrawal , A. Kumar , and S. Gupta , “Bidirectional Effect of Triphala on Modulating Gut‐Brain Axis to Improve Cognition in the Murine Model of Alzheimer's Disease,” Molecular Nutrition & Food Research 68, no. 13 (2024): e2300104. 37767948 10.1002/mnfr.202300104

[130] G. Lamichhane , J. Liu , S. J. Lee , D. Y. Lee , G. Zhang , and Y. Kim , “Curcumin Mitigates the High‐Fat High‐Sugar Diet‐Induced Impairment of Spatial Memory, Hepatic Metabolism, and the Alteration of the Gut Microbiome in Alzheimer's Disease‐Induced (3xTg‐AD) Mice,” Nutrients 16, no. 2 (2024): 240. 38257133 10.3390/nu16020240 PMC10818691

[131] X. Deng , Z. Li , J. Tian , et al., “ *Phyllanthus emblica* L. Polyphenols Mitigate High‐Fat Diet‐Induced Cognitive Impairment via Modulating Gut Microbiota and Calcium/cAMP Signaling Pathways,” Food Bioscience 81 (2026): 109097.

[132] L. J. Chen , S. Sha , H. Stocker , H. Brenner , and B. Schöttker , “The Associations of Serum Vitamin D Status and Vitamin D Supplements Use With All‐Cause Dementia, Alzheimer's Disease, and Vascular Dementia: A UK Biobank Based Prospective Cohort Study,” American Journal of Clinical Nutrition 119, no. 4 (2024): 1052–1064. 38296029 10.1016/j.ajcnut.2024.01.020 PMC11007746

[133] X. X. Zhang , H. R. Wang , Meng‐Wei , et al., “Association of Vitamin D Levels With Risk of Cognitive Impairment and Dementia: A Systematic Review and Meta‐Analysis of Prospective Studies,” Journal of Alzheimer's Disease 98, no. 2 (2024): 373–385. 10.3233/JAD-231381 38461506

[134] S. S. Navale , A. Mulugeta , A. Zhou , et al., “Vitamin D and Brain Health: An Observational and Mendelian Randomization Study,” American Journal of Clinical Nutrition 116, no. 2 (2022): 531–540. 35451454 10.1093/ajcn/nqac107 PMC9348994

[135] N. I. Ely Arman and S. Makpol , “The Link Between Gut Microbiome, Amyloid‐Beta Deposition, Brain Inflammation, and Alzheimer's Disease: A Review of Current Literature,” Current Neuropharmacology (2026), 10.2174/011570159X416900251207210728. 41863277

[136] B. S. Goncalves , N. L. Gannavaram , C. Yates , et al., “Nutritional Strategies Against Dementia in Rural Populations,” Frontiers in Nutrition 12 (2025): 1677197. 41473190 10.3389/fnut.2025.1677197 PMC12745284

[137] U. Shabbir , H. McNulty , C. Hughes , et al., “B Vitamins, Immune Function and the Ageing Brain: A Critical Review of the Evidence, Mechanisms and Potential Role of the Gut Microbiome,” Proceedings of the Nutrition Society (2026): 1–12, 10.1017/S0029665126102249. 41693429

[138] S. Skawratananond , G. E. McCrea , P. Lie , et al., “The Synergistic Interplay Between Vitamin A, Dietary Fiber, and the Microbiota‐Gut‐Brain Axis: A Potential Mechanism for Preventing Alzheimer's Disease,” American Journal of Physiology, Gastrointestinal and Liver Physiology 329, no. 3 (2025): G484–G499. 40796226 10.1152/ajpgi.00097.2025 PMC12622890

[139] Z. L. Wang , S. J. Pang , K. W. Zhang , P. Y. Li , P. G. Li , and C. Yang , “Dietary Vitamin A Modifies the Gut Microbiota and Intestinal Tissue Transcriptome, Impacting Intestinal Permeability and the Release of Inflammatory Factors, Thereby Influencing Aβ Pathology,” Frontiers in Nutrition 11 (2024): 1367086. 38606018 10.3389/fnut.2024.1367086 PMC11008281

[140] B. W. Chen , K. W. Zhang , S. J. Chen , C. Yang , and P. G. Li , “Vitamin A Deficiency Exacerbates Gut Microbiota Dysbiosis and Cognitive Deficits in Amyloid Precursor Protein/Presenilin 1 Transgenic Mice,” Frontiers in Aging Neuroscience 13 (2021): 753351. 34790112 10.3389/fnagi.2021.753351 PMC8591312

[141] C. X. Li , G. R. Cheng , X. C. Liu , et al., “Serum Homocysteine, Hemoglobin, and Alzheimer's Biomarkers Involved in the Relationship of Folate and Vitamin B12 With Cognitive Function: Findings From the Hubei Memory and Aging Cohort Study,” Nutrition Research 149 (2026): 178–188. 41980534 10.1016/j.nutres.2026.03.008

[142] S. Park , S. Kang , and D. Sol Kim , “Folate and Vitamin B‐12 Deficiencies Additively Impaire Memory Function and Disturb the Gut Microbiota in Amyloid‐β Infused Rats,” International Journal for Vitamin and Nutrition Research 92, no. 3–4 (2022): 169–181. 31841076 10.1024/0300-9831/a000624

[143] S. Li , P. Yang , X. Cai , M. He , Y. He , and F. He , “Vitamin C Supplementation Mitigates Mild Cognitive Impairment in Mice Subjected to D‐Galactose: Insights Into Intestinal Flora and Derived SCFAs,” European Journal of Pharmacology 1001 (2025): 177787. 40449647 10.1016/j.ejphar.2025.177787

[144] J. Tynkkynen , V. Chouraki , S. J. van der Lee , et al., “Association of Branched‐Chain Amino Acids and Other Circulating Metabolites With Risk of Incident Dementia and Alzheimer's Disease: A Prospective Study in Eight Cohorts,” Alzheimer's & Dementia 14, no. 6 (2018): 723–733. 10.1016/j.jalz.2018.01.003 PMC6082422 29519576

[145] H. Sato , Y. Takado , S. Toyoda , et al., “Neurodegenerative Processes Accelerated by Protein Malnutrition and Decelerated by Essential Amino Acids in a Tauopathy Mouse Model,” Science Advances 7, no. 43 (2021): eabd5046. 34678069 10.1126/sciadv.abd5046 PMC8535828

[146] S. Pan , Y. Zhang , T. Ye , et al., “A High‐Tryptophan Diet Alleviated Cognitive Impairment and Neuroinflammation in APP/PS1 Mice Through Activating Aryl Hydrocarbon Receptor via the Regulation of Gut Microbiota,” Molecular Nutrition & Food Research 68, no. 2 (2024): e2300601. 38031265 10.1002/mnfr.202300601

[147] X. Wang , G. Sun , T. Feng , et al., “Sodium Oligomannate Therapeutically Remodels Gut Microbiota and Suppresses Gut Bacterial Amino Acids‐Shaped Neuroinflammation to Inhibit Alzheimer's Disease Progression,” Cell Research 29, no. 10 (2019): 787–803. 31488882 10.1038/s41422-019-0216-x PMC6796854

[148] Q. Guo , Y. He , T. Cao , et al., “Tryptophan‐Kynurenine Metabolism: A Link Between the Gut Microbiota Dysbiosis and Cognitive Impairment,” Microbiological Research 307 (2026): 128481. 41764851 10.1016/j.micres.2026.128481

[149] A. Salminen , “Activation of Aryl Hydrocarbon Receptor (AhR) in Alzheimer's Disease: Role of Tryptophan Metabolites Generated by Gut Host‐Microbiota,” Journal of Molecular Medicine 101, no. 3 (2023): 201–222. 36757399 10.1007/s00109-023-02289-5 PMC10036442

[150] H. M. Al‐kuraishy , T. J. Al‐Maiahy , G. M. Sulaiman , et al., “The Potential Role of Aryl Hydrocarbon Receptor in Alzheimer's Disease: Protective or Detrimental,” Ageing Research Reviews 110 (2025): 102810. 40553978 10.1016/j.arr.2025.102810

[151] B. Wang , M. Pan , L. Yang , et al., “ *Akkermansia muciniphila* Reduces Neuroinflammation and Aβ Deposition via Tryptophan Metabolism in the APP/PS1 Mouse Model of Alzheimer's Disease,” Alzheimer's Research & Therapy 18, no. 1 (2026): 41. 10.1186/s13195-025-01880-x PMC12922204 41715194

[152] Y. Xu , L. Huang , L. Sun , et al., “Gut Lachnospiraceae Improves White Matter Injury‐Related Cognitive Decline by Increasing L‐Arginine,” Cellular & Molecular Biology Letters 31, no. 1 (2026): 39. 41724956 10.1186/s11658-026-00866-3 PMC13032417

[153] Y. Zhang , T. Liu , F. Pan , et al., “Dietary Methionine Restriction Alleviates Cognitive Impairment in Alzheimer's Disease Mice via Sex‐Dependent Modulation on Gut Microbiota and Tryptophan Metabolism: A Multiomics Analysis,” Journal of Agricultural and Food Chemistry 73, no. 2 (2025): 1356–1372. 39745486 10.1021/acs.jafc.4c09878

[154] R. Yu , H. Zhang , R. Chen , et al., “Fecal Microbiota Transplantation From Methionine‐Restricted Diet Mouse Donors Improves Alzheimer's Learning and Memory Abilities Through Short‐Chain Fatty Acids,” Food 14, no. 1 (2025): 101. 10.3390/foods14010101 PMC11720665 39796390

[155] H. Liu , N. Zheng , Z. Zhang , et al., “Methionine Restriction Inhibits the TGF‐β1/CCN2/NF‐κB Pathway to Attenuate Astrocyte Inflammation and Cognitive Impairment in the APP/PS1 Mice,” International Immunopharmacology 166 (2025): 115498. 40957174 10.1016/j.intimp.2025.115498

[156] Y. Yang , M. Lu , Y. Xu , J. Qian , G. le , and Y. Xie , “Dietary Methionine via Dose‐Dependent Inhibition of Short‐Chain Fatty Acid Production Capacity Contributed to a Potential Risk of Cognitive Dysfunction in Mice,” Journal of Agricultural and Food Chemistry 70, no. 48 (2022): 15225–15243. 36413479 10.1021/acs.jafc.2c04847

[157] Y. Wang , X. Rong , H. Guan , et al., “The Potential Effects of Isoleucine Restricted Diet on Cognitive Impairment in High‐Fat‐Induced Obese Mice via Gut Microbiota‐Brain Axis,” Molecular Nutrition & Food Research 67, no. 20 (2023): e2200767. 37658490 10.1002/mnfr.202200767

[158] Y. Zhang , X. Li , G. Huang , et al., “Propionate Stimulates the Secretion of Satiety Hormones and Reduces Acute Appetite in a Cecal Fistula Pig Model,” Animal Nutrition 10 (2022): 390–398. 35949198 10.1016/j.aninu.2022.06.003 PMC9356018

[159] J. Liu , J. Cao , L. Jia , et al., “Impacts of Host Genetics on Gut Microbiome Composition in Alzheimer's Disease,” Microbiome 14, no. 1 (2026): 115. 41782023 10.1186/s40168-026-02342-8 PMC13069797

[160] D. M. S. Dissanayaka , T. N. Jayasinghe , H. R. Sohrabi , et al., “Gut Microbial Composition and Short‐Chain Fatty Acid Metabolism in Cognitively Unimpaired Adults Stratified by Amyloid‐β Status,” Biomolecules 16, no. 1 (2025): 18. 41594560 10.3390/biom16010018 PMC12838975

[161] J. F. Kuehn , Q. Zhang , M. B. Heston , et al., “Fecal Short‐Chain Fatty Acids Vary by Sex and Amyloid Status,” medRxiv (2025). 10.1002/alz.70877 PMC12639406 41273231

[162] N. Govindarajan , R. C. Agis‐Balboa , J. Walter , F. Sananbenesi , and A. Fischer , “Sodium Butyrate Improves Memory Function in an Alzheimer's Disease Mouse Model When Administered at an Advanced Stage of Disease Progression,” Journal of Alzheimer's Disease 26, no. 1 (2011): 187–197. 10.3233/JAD-2011-110080 21593570

[163] L. Ho , K. Ono , M. Tsuji , P. Mazzola , R. Singh , and G. M. Pasinetti , “Protective Roles of Intestinal Microbiota Derived Short Chain Fatty Acids in Alzheimer's Disease‐Type Beta‐Amyloid Neuropathological Mechanisms,” Expert Review of Neurotherapeutics 18, no. 1 (2018): 83–90. 29095058 10.1080/14737175.2018.1400909 PMC5958896

[164] C. Nastasi , M. Candela , C. M. Bonefeld , et al., “The Effect of Short‐Chain Fatty Acids on Human Monocyte‐Derived Dendritic Cells,” Scientific Reports 5, no. 1 (2015): 16148. 26541096 10.1038/srep16148 PMC4635422

[165] D. J. Morrison and T. Preston , “Formation of Short Chain Fatty Acids by the Gut Microbiota and Their Impact on Human Metabolism,” Gut Microbes 7, no. 3 (2016): 189–200. 26963409 10.1080/19490976.2015.1134082 PMC4939913

[166] H. Chen , L. Meng , and L. Shen , “Multiple Roles of Short‐Chain Fatty Acids in Alzheimer Disease,” Nutrition 93 (2022): 111499. 34735921 10.1016/j.nut.2021.111499

[167] E. E. Blaak , E. E. Canfora , S. Theis , et al., “Short Chain Fatty Acids in Human Gut and Metabolic Health,” Beneficial Microbes 11, no. 5 (2020): 411–455. 32865024 10.3920/BM2020.0057

[168] J. Tan , C. McKenzie , M. Potamitis , A. N. Thorburn , C. R. Mackay , and L. Macia , “The Role of Short‐Chain Fatty Acids in Health and Disease,” Advances in Immunology 121 (2014): 91–119. 24388214 10.1016/B978-0-12-800100-4.00003-9

[169] Z. Chen , J. Luo , J. Li , et al., “Foxo1 Controls Gut Homeostasis and Commensalism by Regulating Mucus Secretion,” Journal of Experimental Medicine 218, no. 9 (2021): e20210324. 34287641 10.1084/jem.20210324 PMC8424467

[170] R. Vemuri , R. Gundamaraju , T. Shinde , et al., “ *Lactobacillus acidophilus* DDS‐1 Modulates Intestinal‐Specific Microbiota, Short‐Chain Fatty Acid and Immunological Profiles in Aging Mice,” Nutrients 11, no. 6 (2019): 1297. 31181695 10.3390/nu11061297 PMC6627711

[171] P. M. Smith , M. R. Howitt , N. Panikov , et al., “The Microbial Metabolites, Short‐Chain Fatty Acids, Regulate Colonic Treg Cell Homeostasis,” Science 341, no. 6145 (2013): 569–573. 23828891 10.1126/science.1241165 PMC3807819

[172] V. De Preter , K. P. Geboes , V. Bulteel , et al., “Kinetics of Butyrate Metabolism in the Normal Colon and in Ulcerative Colitis: The Effects of Substrate Concentration and Carnitine on the β‐Oxidation Pathway,” Alimentary Pharmacology & Therapeutics 34, no. 5 (2011): 526–532. 21707682 10.1111/j.1365-2036.2011.04757.x

[173] A. Fukushima , Y. Aizaki , and K. Sakuma , “Short‐Chain Fatty Acids Increase the Level of Calbindin‐D9k Messenger RNA in Caco‐2 Cells,” Journal of Nutritional Science and Vitaminology 58, no. 4 (2012): 287–291. 23132313 10.3177/jnsv.58.287

[174] V. Braniste , M. Al‐Asmakh , C. Kowal , et al., “The Gut Microbiota Influences Blood‐Brain Barrier Permeability in Mice,” Science Translational Medicine 6, no. 263 (2014): 263ra158. 10.1126/scitranslmed.3009759 PMC4396848 25411471

[175] R. Corrêa‐Oliveira , J. L. Fachi , A. Vieira , F. T. Sato , and M. A. Vinolo , “Regulation of Immune Cell Function by Short‐Chain Fatty Acids,” Clinical & Translational Immunology 5, no. 4 (2016): e73. 27195116 10.1038/cti.2016.17 PMC4855267

[176] B. Dalile , L. Van Oudenhove , B. Vervliet , et al., “The Role of Short‐Chain Fatty Acids in Microbiota‐Gut‐Brain Communication,” Nature Reviews Gastroenterology & Hepatology 16, no. 8 (2019): 461–478. 31123355 10.1038/s41575-019-0157-3

[177] D. M. S. Dissanayaka , T. N. Jayasinghe , H. R. Sohrabi , et al., “Functional Pathways of the Gut Microbiome Associated With SCFA Profiles in Preclinical Alzheimer's Disease,” Aging and Disease (2026), 10.14336/AD.2025.1539. 41619271

[178] A. Martínez‐del C , S. Bodea , H. A. Hamer , et al., “Characterization and Detection of a Widely Distributed Gene Cluster That Predicts Anaerobic Choline Utilization by Human Gut Bacteria,” MBio 6, no. 2 (2015): e00042‐15. 25873372 10.1128/mBio.00042-15 PMC4453576

[179] P. Gatarek and J. Kaluzna‐Czaplinska , “Trimethylamine N‐Oxide (TMAO) in Human Health,” EXCLI Journal 20 (2021): 301–319. 33746664 10.17179/excli2020-3239 PMC7975634

[180] G. N. Wiese , A. Biruete , R. N. Moorthi , S. M. Moe , S. R. Lindemann , and K. M. Hill Gallant , “Plant‐Based Diets, the Gut Microbiota, and Trimethylamine N‐Oxide Production in Chronic Kidney Disease: Therapeutic Potential and Methodological Considerations,” Journal of Renal Nutrition 31, no. 2 (2021): 121–131. 32616440 10.1053/j.jrn.2020.04.007 PMC7770016

[181] W. Yoo , J. K. Zieba , N. J. Foegeding , et al., “High‐Fat Diet‐Induced Colonocyte Dysfunction Escalates Microbiota‐Derived Trimethylamine N‐Oxide,” Science 373, no. 6556 (2021): 813–818. 34385401 10.1126/science.aba3683 PMC8506909

[182] N. M. Vogt , K. A. Romano , B. F. Darst , et al., “The Gut Microbiota‐Derived Metabolite Trimethylamine N‐Oxide Is Elevated in Alzheimer's Disease,” Alzheimer's Research & Therapy 10, no. 1 (2018): 124. 10.1186/s13195-018-0451-2 PMC6303862 30579367

[183] Z. Ren and L. Mo , “Association Between Levels of Trimethylamine N‐Oxide and Cognitive Dysfunction: A Systematic Review and Meta‐Analysis,” PeerJ 13 (2025): e20000. 40936751 10.7717/peerj.20000 PMC12422268

[184] C. Long , Z. Li , H. Feng , et al., “Association of Trimethylamine Oxide and Its Precursors With Cognitive Impairment: A Systematic Review and Meta‐Analysis,” Frontiers in Aging Neuroscience 16 (2024): 1465457. 39430973 10.3389/fnagi.2024.1465457 PMC11486729

[185] D. Li , Y. Ke , R. Zhan , et al., “Trimethylamine‐N‐Oxide Promotes Brain Aging and Cognitive Impairment in Mice,” Aging Cell 17, no. 4 (2018): e12768. 29749694 10.1111/acel.12768 PMC6052480

[186] R. Xu and Q. Wang , “Towards Understanding Brain‐Gut‐Microbiome Connections in Alzheimer's Disease,” BMC Systems Biology 10, no. Suppl 3 (2016): 63. 27585440 10.1186/s12918-016-0307-y PMC5009560

[187] W. Zhang and Y. Lü , “Trimethylamine‐N‐Oxide Regulates Cognitive Function in Alzheimer's Disease Patients and Mice Model Through Brain‐Gut‐Microbiota Axis,” Alzheimer's & Dementia 20, no. S2 (2024): e086101.

[188] Z. Zhuang , M. Gao , R. Yang , Z. Liu , W. Cao , and T. Huang , “Causal Relationships Between Gut Metabolites and Alzheimer's Disease: A Bidirectional Mendelian Randomization Study,” Neurobiology of Aging 100 (2021): 115–119. 10.1016/j.neurobiolaging.2020.10.022 33280888

[189] L. Hoyles , M. G. Pontifex , I. Rodriguez‐Ramiro , et al., “Regulation of Blood‐Brain Barrier Integrity by Microbiome‐Associated Methylamines and Cognition by Trimethylamine N‐Oxide,” Microbiome 9, no. 1 (2021): 235. 34836554 10.1186/s40168-021-01181-z PMC8626999

[190] G. D. Miller , J. Ragalie‐Carr , and M. Torres‐Gonzalez , “Perspective: Seeing the Forest Through the Trees: The Importance of Food Matrix in Diet Quality and Human Health,” Advances in Nutrition 14, no. 3 (2023): 363–365. 36934833 10.1016/j.advnut.2023.03.005 PMC10201811

[191] S. J. Simpson , D. G. Le Couteur , L. Small , et al., “Macronutrient Mixtures and Interactions in Health and Disease,” Nature Reviews. Endocrinology (2026), 10.1038/s41574-026-01266-5. 42331991

[192] A. Więckowska‐Gacek , A. Mietelska‐Porowska , M. Wydrych , and U. Wojda , “Western Diet as a Trigger of Alzheimer's Disease: From Metabolic Syndrome and Systemic Inflammation to Neuroinflammation and Neurodegeneration,” Ageing Research Reviews 70 (2021): 101397. 34214643 10.1016/j.arr.2021.101397

[193] E. E. Noble , C. A. Olson , E. Davis , et al., “Gut Microbial Taxa Elevated by Dietary Sugar Disrupt Memory Function,” Translational Psychiatry 11, no. 1 (2021): 194. 33790226 10.1038/s41398-021-01309-7 PMC8012713

[194] P. Jiang , W. Zheng , X. Sun , et al., “Sulfated Polysaccharides From *Undaria pinnatifida* Improved High Fat Diet‐Induced Metabolic Syndrome, Gut Microbiota Dysbiosis and Inflammation in BALB/c Mice,” International Journal of Biological Macromolecules 167 (2021): 1587–1597. 33217459 10.1016/j.ijbiomac.2020.11.116

[195] S. G. Koulas , C. K. Stefanou , S. K. Stefanou , et al., “Gut Microbiota in Patients With Morbid Obesity Before and After Bariatric Surgery: A Ten‐Year Review Study (2009‐2019),” Obesity Surgery 31, no. 1 (2021): 317–326. 33130944 10.1007/s11695-020-05074-2

[196] M. Thapa , A. Kumari , C. Y. K. Chin , et al., “Translocation of Bacteria From the Gut to the Brain in Mice,” PLoS Biology 24, no. 3 (2026): e3003652. 41818176 10.1371/journal.pbio.3003652 PMC12981431

[197] H. Shi , X. Ge , X. Ma , et al., “A Fiber‐Deprived Diet Causes Cognitive Impairment and Hippocampal Microglia‐Mediated Synaptic Loss Through the Gut Microbiota and Metabolites,” Microbiome 9, no. 1 (2021): 223. 34758889 10.1186/s40168-021-01172-0 PMC8582174

[198] V. Solfrizzi , C. Custodero , M. Lozupone , et al., “Relationships of Dietary Patterns, Foods, and Micro‐ and Macronutrients With Alzheimer's Disease and Late‐Life Cognitive Disorders: A Systematic Review,” Journal of Alzheimer's Disease 59, no. 3 (2017): 815–849. 10.3233/JAD-170248 28697569

[199] G. Tsivgoulis , S. Judd , A. J. Letter , et al., “Adherence to a Mediterranean Diet and Risk of Incident Cognitive Impairment,” Neurology 80, no. 18 (2013): 1684–1692. 23628929 10.1212/WNL.0b013e3182904f69 PMC3716473

[200] A. C. van den Brink , E. M. Brouwer‐Brolsma , A. Berendsen , et al., “The Mediterranean, Dietary Approaches to Stop Hypertension (DASH), and Mediterranean‐DASH Intervention for Neurodegenerative Delay (MIND) Diets Are Associated With Less Cognitive Decline and a Lower Risk of Alzheimer's Disease – A Review,” Advances in Nutrition 10, no. 6 (2019): 1040–1065. 31209456 10.1093/advances/nmz054 PMC6855954

[201] A. Bach‐Faig , E. M. Berry , D. Lairon , et al., “Mediterranean Diet Pyramid Today. Science and Cultural Updates,” Public Health Nutrition 14, no. 12A (2011): 2274–2284. 22166184 10.1017/S1368980011002515

[202] M. N. K. Fekete , T. S. Jarecsny , A. Lehoczki , et al., “Mediterranean Diet, Polyphenols, and Neuroprotection: Mechanistic Insights Into Resveratrol and Oleuropein,” Nutrients 17, no. 24 (2025): 3929. 41470875 10.3390/nu17243929 PMC12735559

[203] R. Nagpal , C. A. Shively , T. C. Register , et al., “Gut Microbiome‐Mediterranean Diet Interactions in Improving Host Health,” F1000Research 8 (2019): 699. 32704349 10.12688/f1000research.18992.1 PMC7359750

[204] L. J. Appel , T. J. Moore , E. Obarzanek , et al., “A Clinical Trial of the Effects of Dietary Patterns on Blood Pressure. DASH Collaborative Research Group,” New England Journal of Medicine 336, no. 16 (1997): 1117–1124. 9099655 10.1056/NEJM199704173361601

[205] M. C. Morris , C. C. Tangney , Y. Wang , et al., “MIND Diet Slows Cognitive Decline With Aging,” Alzheimer's & Dementia 11, no. 9 (2015): 1015–1022. 10.1016/j.jalz.2015.04.011 PMC4581900 26086182

[206] G. Park , S. Kadyan , N. Hochuli , et al., “A Modified Mediterranean‐Style Diet Enhances Brain Function via Specific Gut‐Microbiome‐Brain Mechanisms,” Gut Microbes 16, no. 1 (2024): 2323752. 38444392 10.1080/19490976.2024.2323752 PMC10936641

[207] A. H. Dilmore , C. Martino , B. J. Neth , et al., “Effects of a Ketogenic and Low‐Fat Diet on the Human Metabolome, Microbiome, and Foodome in Adults at Risk for Alzheimer's Disease,” Alzheimer's & Dementia 19, no. 11 (2023): 4805–4816. 10.1002/alz.13007 PMC10551050 37017243

[208] C. A. Olson , A. J. Iñiguez , G. E. Yang , et al., “Alterations in the Gut Microbiota Contribute to Cognitive Impairment Induced by the Ketogenic Diet and Hypoxia,” Cell Host & Microbe 29, no. 9 (2021): 1378–1392. 34358434 10.1016/j.chom.2021.07.004 PMC8429275

[209] J. Gudden , V. A. Arias , and M. Bloemendaal , “The Effects of Intermittent Fasting on Brain and Cognitive Function,” Nutrients 13, no. 9 (2021): 3166. 34579042 10.3390/nu13093166 PMC8470960

[210] T. Pérez‐Gerdel , M. Camargo , M. Alvarado , and J. D. Ramírez , “Impact of Intermittent Fasting on the Gut Microbiota: A Systematic Review,” Advanced Biology (Weinheim) 7, no. 8 (2023): e2200337. 10.1002/adbi.202200337 36950759

[211] R. Y. Pan , J. Zhang , J. Wang , et al., “Intermittent Fasting Protects Against Alzheimer's Disease in Mice by Altering Metabolism Through Remodeling of the Gut Microbiota,” Nature Aging 2, no. 11 (2022): 1024–1039. 37118092 10.1038/s43587-022-00311-y

[212] F. Cignarella , C. Cantoni , L. Ghezzi , et al., “Intermittent Fasting Confers Protection in CNS Autoimmunity by Altering the Gut Microbiota,” Cell Metabolism 27, no. 6 (2018): 1222–1235. 29874567 10.1016/j.cmet.2018.05.006 PMC6460288

[213] P. Rangan , I. Choi , M. Wei , et al., “Fasting‐Mimicking Diet Modulates Microbiota and Promotes Intestinal Regeneration to Reduce Inflammatory Bowel Disease Pathology,” Cell Reports 26, no. 10 (2019): 2704–2719. 30840892 10.1016/j.celrep.2019.02.019 PMC6528490

[214] M. Guo , X. Wang , Y. Li , et al., “Intermittent Fasting on Neurologic Diseases: Potential Role of Gut Microbiota,” Nutrients 15, no. 23 (2023): 4915. 38068773 10.3390/nu15234915 PMC10707790

[215] C. Xi , J. Zhang , H. Liu , et al., “Can Early Gut Microbiota Screening Reduce the Incidence of Cognitive Impairment? A Mendelian Randomization Study,” Journal of Alzheimer's Disease 102, no. 1 (2024): 195–206. 10.3233/JAD-231457 39497320

[216] Y. Li , H. Lin , K. Liu , and X. Kan , “Human Trace Elements, Gut Microbiota, and Alzheimer's Disease: Insights From Multistage Mendelian Randomization Analysis,” Food Science & Nutrition 13, no. 8 (2025): e70706. 40801051 10.1002/fsn3.70706 PMC12340980

[217] J. Xu , H. Zhang , and J. Jiang , “Precision Nutrition for Management of Cognitive Impairment,” Precision Nutrition 3, no. 4 (2024): e00090.

[218] D. Tuigunov , Y. Sinyavskiy , T. Nurgozhin , et al., “Precision Nutrition and Gut‐Brain Axis Modulation in the Prevention of Neurodegenerative Diseases,” Nutrients 17, no. 19 (2025): 3068. 41097145 10.3390/nu17193068 PMC12526097

[219] N. G. Norwitz , N. Saif , I. E. Ariza , and R. S. Isaacson , “Precision Nutrition for Alzheimer's Prevention in ApoE4 Carriers,” Nutrients 13, no. 4 (2021): 1362. 33921683 10.3390/nu13041362 PMC8073598

[220] K. Ivanich , A. Yackzan , A. Flemister , et al., “Ketogenic Diet Modulates Gut Microbiota‐Brain Metabolite Axis in a Sex‐ and Genotype‐Specific Manner in APOE4 Mice,” Journal of Neurochemistry 169, no. 9 (2025): e70216. 40890565 10.1111/jnc.70216 PMC12870807

[221] S. Clemente‐Velasco , B. de Lucas , M. Tabone , et al., “Study Protocol for Design of a Personalized Dietary Supplement Based on the Gut Microbiota of Alzheimer's Patients and Evaluation of Its Effects in a Pilot Randomized Controlled Trial,” Frontiers in Nutrition 12 (2025): 1653841. 41064285 10.3389/fnut.2025.1653841 PMC12500686

[222] T. J. Jewell , M. Minehan , J. Williams , et al., “Precision Nutrition for Dementia: Exploring the Potential in Mitigating Dementia Progression,” Journal of Dementia and Alzheimer's Disease 2 (2025): 28.

[223] M. N. I. Bhuiyan , B. K. Saha , and M. A. Satter , “Harnessing Artificial Intelligence and Precision Diets for Brain Health and Cognitive Resilience,” Journal of Nutrition 155, no. 10 (2025): 3179–3190. 40812476 10.1016/j.tjnut.2025.08.007

[224] D. Kirk , C. Catal , and B. Tekinerdogan , “Precision Nutrition: A Systematic Literature Review,” Computers in Biology and Medicine 133 (2021): 104365. 33866251 10.1016/j.compbiomed.2021.104365

[225] S. Charisis , M. Yannakoulia , and N. Scarmeas , “Diets to Promote Healthy Brain Ageing,” Nature Reviews. Neurology 21, no. 1 (2025): 5–16. 39572782 10.1038/s41582-024-01036-9

[226] A. Lahiouel , J. Kellett , S. Isbel , and N. M. D'Cunha , “An Exploratory Study of Nutrition Knowledge and Challenges Faced by Informal Carers of Community‐Dwelling People With Dementia: Online Survey and Thematic Analysis,” Geriatrics 8, no. 4 (2023): 77. 37489325 10.3390/geriatrics8040077 PMC10366724

[227] S. Sharma , B. Bashir , K. A. Kolekar , et al., “Tailoring the Biomarkers of Alzheimer's Disease Using a Gut Microbiome‐Centric Approach: Preclinical, Clinical, and Regulatory Perspectives,” Ageing Research Reviews 112 (2025): 102888. 40902672 10.1016/j.arr.2025.102888

[228] A. N. Mafe and D. Büsselberg , “Could a Mediterranean Diet Modulate Alzheimer's Disease Progression? The Role of Gut Microbiota and Metabolite Signatures in Neurodegeneration,” Food 14, no. 9 (2025): 1559. 10.3390/foods14091559 PMC12071848 40361641

[229] J. Sun , J. Xu , Y. Ling , et al., “Fecal Microbiota Transplantation Alleviated Alzheimer's Disease‐Like Pathogenesis in APP/PS1 Transgenic Mice,” Translational Psychiatry 9, no. 1 (2019): 189. 31383855 10.1038/s41398-019-0525-3 PMC6683152

[230] M. S. Kim , Y. Kim , H. Choi , et al., “Transfer of a Healthy Microbiota Reduces Amyloid and Tau Pathology in an Alzheimer's Disease Animal Model,” Gut 69, no. 2 (2020): 283–294. 31471351 10.1136/gutjnl-2018-317431

[231] M. Tian , D. Wang , C. Zhang , et al., “Gut Microbiota Dysbiosis Drives Early Alzheimer's Pathogenesis via Microglial TREM2/SYK/NF‐κB Signaling Axis,” ACS Chemical Neuroscience 17, no. 11 (2026): 2190–2202. 42130461 10.1021/acschemneuro.6c00173

[232] F. Valeri , M. Dos Santos Guilherme , F. He , et al., “Impact of the Age of Cecal Material Transfer Donors on Alzheimer's Disease Pathology in 5xFAD Mice,” Microorganisms 9, no. 12 (2021): 2548. 34946148 10.3390/microorganisms9122548 PMC8708188

[233] C. Chevalier , B. B. Tournier , M. Marizzoni , et al., “Fecal Microbiota Transplantation (FMT) From a Human at Low Risk for Alzheimer's Disease Improves Short‐Term Recognition Memory and Increases Neuroinflammation in a 3xTg AD Mouse Model,” Genes, Brain, and Behavior 24, no. 1 (2025): e70012. 39801363 10.1111/gbb.70012 PMC11725982

[234] X. Jiang , Y. Zheng , H. Sun , et al., “Fecal Microbiota Transplantation Improves Cognitive Function of a Mouse Model of Alzheimer's Disease,” CNS Neuroscience & Therapeutics 31, no. 2 (2025): e70259. 39957504 10.1111/cns.70259 PMC11831070

[235] S. Soriano , A. Marshall , M. Holcomb , et al., “Sex‐Specific Effects of Fecal Microbiota Transplantation on TBI‐Exacerbated Alzheimer's Disease Pathology in Mice,” Frontiers in Microbiology 16 (2025): 1703708. 41704851 10.3389/fmicb.2025.1703708 PMC12907400

[236] S. Alaeddin , A. Chatterjee , T. L. Roberts , et al., “Exploring the Effects of Faecal Microbiota Transplantation on Cognitive Function: A Review of Clinical Trials,” Brain, Behavior and Immunity ‐ Health 48 (2025): 101049. 10.1016/j.bbih.2025.101049 PMC12273246 40686934

[237] X. Chen , W. Zhang , Z. Lin , et al., “Preliminary Evidence for Developing Safe and Efficient Fecal Microbiota Transplantation as Potential Treatment for Aged Related Cognitive Impairments,” Frontiers in Cellular and Infection Microbiology 13 (2023): 1103189. 37113132 10.3389/fcimb.2023.1103189 PMC10127103

[238] S. Hazan , “Rapid Improvement in Alzheimer's Disease Symptoms Following Fecal Microbiota Transplantation: A Case Report,” Journal of International Medical Research 48, no. 6 (2020): 300060520925930. 32600151 10.1177/0300060520925930 PMC7328362

[239] S. H. Park , J. H. Lee , J. Shin , et al., “Cognitive Function Improvement After Fecal Microbiota Transplantation in Alzheimer's Dementia Patient: A Case Report,” Current Medical Research and Opinion 37, no. 10 (2021): 1739–1744. 34289768 10.1080/03007995.2021.1957807

[240] W. Xiang , H. Xiang , J. Wang , et al., “Fecal Microbiota Transplantation: A Novel Strategy for Treating Alzheimer's Disease,” Frontiers in Microbiology 14 (2023): 1281233. 38033557 10.3389/fmicb.2023.1281233 PMC10687436

[241] J. Ren , Q. Wang , H. Hong , and C. Tang , “Fecal Microbiota Transplantation in Alzheimer's Disease: Mechanistic Insights Through the Microbiota‐Gut‐Brain Axis and Therapeutic Prospects,” Microorganisms 13, no. 8 (2025): 1956. 40871460 10.3390/microorganisms13081956 PMC12388133

[242] M. Maor , H. Levy Barazany , and I. Kolodkin‐Gal , “The Ladder of Regulatory Stringency and Balance: An Application to the US FDA's Regulation of Bacterial Live Therapeutics,” Gut Microbes 17, no. 1 (2025): 2517377. 40501442 10.1080/19490976.2025.2517377 PMC12164374

[243] Z. Zou , Y. Yin , B. Liao , Z. Lu , M. Su , and X. Li , “Fecal Microbiota Transplantation for Alzheimer's Disease: A Review of Current Evidence and Future Perspectives,” Current Clinical Microbiology Reports 12, no. 1 (2025): 14.

[244] J. Zhao , Y. Fan , K. Yang , and H. Gao , “Fecal Microbiota Transplantation: From Empirical Remedy to Precision Medicine,” Frontiers in Microbiomes 5 (2026): 1863308. 42388392 10.3389/frmbi.2026.1863308 PMC13318992

[245] B. Salman , R. Haidar , V. Faour , et al., “Microbiota Gut‐Brain Axis Dysfunction in Alzheimer's Disease: From Pathogenesis to Potential Treatments – A Review,” Health Sciences Review 18 (2026): 100251.

[246] A. S. James , N. A. Adil , D. Goltz , et al., “Abnormalities in Gut Virome Signatures Linked With Cognitive Impairment in Older Adults,” Gut Microbes 16, no. 1 (2024): 2431648. 39676708 10.1080/19490976.2024.2431648 PMC11651276

[247] M. Li , H. Yang , C. Shao , Y. Liu , S. Wen , and L. Tang , “Application of Dominant Gut Microbiota Promises to Replace Fecal Microbiota Transplantation as a New Treatment for Alzheimer's Disease,” Microorganisms 11, no. 12 (2023): 2854. 38137998 10.3390/microorganisms11122854 PMC10745325

[248] P. Feng , W. Zhang , Y. Zhao , P. Zhao , and E. Li , “Synthetic Microbial Communities: A Novel Emerging Models for Dissecting Gut Microbiota‐Host Interactions in Neurodegenerative Diseases,” Frontiers in Immunology 17 (2026): 1822743. 42253968 10.3389/fimmu.2026.1822743 PMC13233190

[249] B. Zhang , Z. Sun , W. Song , H. Huang , and Y. Wu , “Engineered Butyrate‐Producing Yeasts Mitigate Alzheimer‐Associated Phenotypes,” Signal Transduction and Targeted Therapy 10, no. 1 (2025): 369. 41242996 10.1038/s41392-025-02474-7 PMC12620515

[250] H. Sutanto and D. Fetarayani , “Engineering the Gut Microbiome: Synthetic Biology Approaches for Human Health and Disease,” Gut Microbiology 2 (2026): 100005.

